# Zooplankton carcasses stimulate microbial turnover of allochthonous particulate organic matter

**DOI:** 10.1038/s41396-020-00883-w

**Published:** 2021-01-18

**Authors:** Darshan Neubauer, Olesya Kolmakova, Jason Woodhouse, Robert Taube, Kai Mangelsdorf, Michail Gladyshev, Katrin Premke, Hans-Peter Grossart

**Affiliations:** 1 Leibniz Institute of Freshwater Ecology and Inland Fisheries (IGB), Department of Experimental Limnology, 16775 Stechlin, Germany; 2 Institute of Biochemistry and Biology, Potsdam University, 14476 Potsdam, Germany; 3 Institute of Biophysics SB RAS, Federal Research Center “Krasnoyarsk Science Center SB RAS”, Krasnoyarsk, Russia; 4 Siberian Federal University, Institute of Fundamental Biology and Biotechnology, Krasnoyarsk, Russia; 5 GFZ German Research Centre -for Geosciences, Helmholtz Centre Potsdam, Section 3.2 Organic Geochemistry, 14473 Potsdam, Germany; 6 Leibniz Institute of Freshwater Ecology and Inland Fisheries (IGB), Department of Chemical Analytics and Biogeochemistry, Müggelseedamm 310, 12587 Berlin, Germany

**Keywords:** Limnology, Biogeochemistry, Ecology, Microbiology

## Abstract

Carbon turnover in aquatic environments is dependent on biochemical properties of organic matter (OM) and its degradability by the surrounding microbial community. Non-additive interactive effects represent a mechanism where the degradation of biochemically persistent OM is stimulated by the provision of bioavailable OM to the degrading microbial community. Whilst this is well established in terrestrial systems, whether it occurs in aquatic ecosystems remains subject to debate. We hypothesised that OM from zooplankton carcasses can stimulate the degradation of biochemically persistent leaf material, and that this effect is influenced by the daphnia:leaf OM ratio and the complexity of the degrading microbial community. Fresh *Daphnia magna* carcasses and ^13^C-labelled maize leaves (*Zea mays)* were incubated at different ratios (1:1, 1:3 and 1:5) alongside either a complex microbial community (<50 µm) or solely bacteria (<0.8 µm). ^13^C stable-isotope measurements of CO_2_ analyses were combined with phospholipid fatty acids (PLFA) analysis and DNA sequencing to link metabolic activities, biomass and taxonomic composition of the microbial community. Our experiments indicated a significantly higher respiration of leaf-derived C when daphnia-derived OM was most abundant (i.e. daphnia:leaf OM ratio of 1:1). This process was stronger in a complex microbial community, including eukaryotic microorganisms, than a solely bacterial community. We concluded that non-additive interactive effects were a function of increased C–N chemodiversity and microbial complexity, with the highest net respiration to be expected when chemodiversity is high and the degrading community complex. This study indicates that identifying the interactions and processes of OM degradation is one important key for a deeper understanding of aquatic and thus global carbon cycle.

## Introduction

Freshwaters are seasonally dynamic ecosystems that play an active role in biogeochemical cycles, such as organic matter (OM) sequestration or remineralisation. Even if lakes only make a small fraction of the global carbon (C) budget [1, 2], they are active ecosystems that transport, transform and store considerable amounts of C, and therefore have a significant effect on the global C cycle [3]. Thus, identifying interactions and processes impacting C fluxes is an important step towards a better understanding of global C cycling [4]. The C pool of aquatic environments is composed of autochthonous OM, such as algae, zooplankton and macrophytes, and terrestrially derived (i.e. allochthonous) OM, such as leaves and leaf leachate from the catchment area. Seasonally changing sources of OM (e.g., algae blooms, zooplankton successions, pollen deposition or litter fall) provide freshwaters with a continuously changing diversity of OM [5]. Aquatic OM turnover is mainly controlled by heterotrophic microbes, efficiently exploiting C and nutrients from different sources to meet their energy and growth demands [6–8].

The rates of microbial OM turnover depend on OM quantity and on the biochemical properties (e.g., molecular weight, chemical structure (monomeric, polymeric and colloidal) and stoichiometry) of the specific organic carbon compounds [9–11]. Autochthonous OM consists predominantly of low-molecular-weight compounds, including simple carbohydrates, amino acids and acetate [12]. Thus, autochthonous OM is generally easily accessible to microorganisms (i.e. bioavailable) ensuring a short turnover time in the environment [13]. In contrast, allochthonous OM (e.g., leaf litter) has a large fraction of insoluble, complex polymeric carbohydrates, such as lignin (15–40%), cellulose (10–50%) and hemicelluloses (30–40%) [14], which require specific, energetically expensive enzymes for microbial utilisation [15, 16]. Consequently, the insoluble fraction of terrestrially derived OM (i.e. mainly lignocellulose derivatives in leaf litter) accumulates in aquatic environments, forming a large pool of allochthonous OM [12]. Phytoplankton blooms, zooplankton successions and litter fall contribute to the seasonal dynamics of allochthonous and autochthonous OM pools in freshwater ecosystems and thus impact microbial auto- and heterotrophic activity [17]. This results in a complex series of cascading and partly unknown effects on OM bioavailability and chemodiversity influencing net C fluxes [18].

It has been hypothesised that an increase in chemodiversity, e.g., an addition of bioavailable OM to a system, provides the microbial community with additional energy and affects the net breakdown of biochemically more persistent OM, a concept termed the “priming effect” [19, 20]. Because priming effects can be positive (enhanced degradation of less bioavailable material upon the addition of easily accessible OM) or negative (decreased degradation of the less bioavailable fraction due to a preferential consumption of the more bioavailable OM), Bengtsson et al. [21] suggested to use the term “interactive effects” over “priming effect”, to avoid biasing the interpretation of results a priori. While the concept of interactive effects is widely accepted in soil sciences [22], its impact on net OM turnover in aquatic ecosystems is not obvious [21, 23–25]. Two meta-analyses concluded that interactive effects do not significantly increase net OM degradation rates in freshwater environments [21, 24]. The authors urge on the need to acknowledge microbial diversity and the range of different molecular compounds included in distinct OM sources [21], and to consider nutrient availability and stoichiometry as drivers of interactive effects [24]. Although numerous studies have evidenced interactive effects at the terrestrial–aquatic interface [26–29], there is little consensus about the role of interactive effects on net OM turnover, perhaps because of the lack of mechanistic understanding to predict where and when they can be expected [23].

Zooplankton carcasses are an abundant but poorly studied OM source in freshwaters [30]. While the exuviae of daphnids consists of relatively persistent chitin (3–7% of total daphnia biomass), most of the biomass comprises proteins (30% upward), lipids (up to 20%) and carbohydrates (10–30%) and hence represents a mainly easy-to-degrade and bioavailable OM source [31, 32]. Zooplankton experiences non-predatory mortality, resulting from pathogen infections, starvation or environmental stress [30, 33], with a significant fraction of carcasses persisting for several days in the water column before sedimentation [34]. Zooplankton carcasses represent hotspots of OM turnover, providing the microbial community with a growth structure (i.e., the chitin exuviae) facilitating attachment and colonisation, and a diversity of nutrient-rich OM compounds [32, 35, 36]. Seasonal fluctuations in zooplankton-derived OM trigger shifts in microbial community composition [37], but whether it can stimulate the degradation of less bioavailable, leaf-derived OM remains unknown.

Eukaryotic microorganisms (e.g., fungi, oomycetes and protozoa) play an important role in the degradation of terrestrially derived OM and stimulate the turnover of OM [10, 38, 39]. Fungi are well-characterised degraders of various terrestrially derived OM, via the production of extracellular ligninolytic oxidative enzymes, which can physically penetrate and break down colloidal and particulate OM [40–42]. Although the microbial degradation of lignocellulose has been intensively studied in fungi [15, 39, 43], there is emerging evidence that bacteria are involved in delignification [44, 45]. Protozoa do not directly consume particulate OM or complex macromolecules, like lignocellulose derivatives, yet, by grazing on bacteria and excreting secondary metabolites they have significant effects on the bacterial community structure, the composition of the C and nutrient pools and, consecutively, on net degradation rates of OM [38, 46]. Therefore, the complexity of microbial communities and interactions should be considered when studying aquatic OM turnover and C fluxes.

Using a stable-isotope and sequencing approach, we incubated a complex microbial lake community and a simplified microbial (mostly small bacteria) community with *Daphnia magna* carcasses (acting as a predominantly bioavailable OM pool) and *Zea mays*-derived lignocellulose (as a biochemically persistent OM pool) in different concentrations. We selected a gradient of daphnia- to leaf-derived OM ratios (1:1, 1:3 and 1:5), to simulate interactive effects occurring at different seasonal states [30, 33, 34]. These range from high zooplankton-derived OM abundances during the clear-water phase (i.e., 1:1 ratio) to lower concentrations (i.e., ratios of 1:3 and 1:5) resulting from the subsequent decline of zooplankton populations. The maize leaves were ^13^C-labelled to trace its microbial mineralisation via ^13^CO_2_ measurements and microbial assimilation by membrane phospholipid fatty acid (PLFA) analysis. PLFAs, found in cell membranes of all living organisms, rapidly degrade upon cell death [47], and therefore serve as an indicator for living biomass. Isotope analyses of PLFAs follow the assimilation of C from different substrates (distinct in their isotopic signature) into the biomass of different microbial groups (e.g., eukaryotic vs. prokaryotic). Based on previous findings [10, 38, 48], we expect that a complex microbial community (including eukaryotic microorganisms and bacteria) will exploit leaf-derived C more efficiently compared to a simplified, bacteria-dominated community. We hypothesised that a complex and chemodiverse system combining both, a predominantly bioavailable source of OM (i.e., zooplankton carcasses) and a rather persistent OM source (i.e., maize leaf-derived lignocellulose), would lead to a higher utilisation of the persistent OM source. Furthermore, we examined how seasonal dynamics of zooplankton OM availability, reflected by the ratios of daphnia- to leaf-derived OM (D:L), influence the direction and magnitude of interactive effects. Zooplankton-derived OM is often overlooked as it is short-lived and comprises only a small proportion of the total OM pool. However, mass mortality events, much like phytoplankton blooms, can result in drastic shifts in OM composition and stimulate microbial activity which, in the short term, promotes the turnover of persistent OM.

## Methods

### Experimental setup

Incubated microbial communities originated from the north-eastern (NE), less acidic basin of Lake Große Fuchskuhle (Northeastern Germany, 53°06′N 12°59′E). This bog lake receives large amounts of terrestrially derived OM and its microbial community is therefore adapted to persistent terrestrial OM [49]. The water for inoculum preparation was sampled from the shore, where the microbial community is in close contact with the surrounding accumulated leaf litter and, thus, expected to be well adapted to leaf-litter degradation. Two inoculum solutions were prepared: one with a complex microbial community (including eukaryotic microorganisms like protozoa and fungi) and another with a solely bacterial community (see protocol in the Supplementary). Incubations were performed on a roller apparatus in the dark at temperatures fluctuating between 16 and 18 °C. One litre acid-washed and muffled glass bottles were filled (air-bubble free) with artificial acidic lake water [50] adjusted to the pH of the NE basin of Lake Große Fuchskuhle (i.e., 5.7) and inoculated with 1 ml of bacterial or total microbial community concentrate accordingly. We estimated the initial cell density in the total microbial community treatments to be in the range of 2.5 × 10^6^ cells ml^−1^ and roughly 10^6^ cells ml^−1^ in the bacterial treatment (see Supplementary Information for calculations). Even though these estimates are lower than natural bacterial concentrations in Lake Große Fuchskuhle (~5 × 10^7^ cells ml^−1^), they are still in the range of natural bacterial densities.

Cultured *Daphnia magna* was freeze-dried, to allow a precise determination of the added dry weight, and used as a source for zooplankton-derived OM. We selected maize leaves (*Zea mays*, ~10 atom% ^13^C, IsoLife bv) as a model substrate for lignocellulose derivatives, which are the predominating C compounds in leaf litter and the ^13^C enrichment makes it possible to determine its specific utilisation. The maize leaves were shredded and leached for 48 h in the dark in distilled water at room temperature to remove all soluble compounds [51], and ensure that the remaining leaf material consisted mainly of insoluble lignocellulosic compounds. All treatments were amended with the 6 mg l^−1^ of OM, but using three different daphnia- to leaf-derived OM ratios: 1:1, 1:3 and 1:5 (see Table 1). Three additional treatments with only daphnia, only leaves and a blank served as controls. For each treatment, four initial replicates (*n*_initials_ = 4), four final replicates (*n*_finals_ = 4) and one additional bottle for daily monitoring were prepared, sealed with PTFE-coated silicone septa and placed horizontally on a roller apparatus at the above-mentioned incubating conditions. The four initial replicates were sacrificed on the same day to measure initial values. The daily monitoring bottles served to measure dissolved gas concentrations (i.e., O_2_, ^12^CO_2_ and ^13^CO_2_) to ensure that the incubations remained oxic and to follow the daily microbial utilisation of daphnia- and leaf-derived OM.

**Table 1:** Overview on the different treatments.

Treatment	b-DL1:1	DL1:1	DL1:3	DL1:5	Daphnia	Leaves	Blank
Inoculum	Bacterial community < 0.8 µm	Complex microbial community < 50 µm					
Daphnia:leaves ratio	1:1	1:1	1:3	1:5	Controls		
Daphnia (mg l^−1^)	3	3	1.5	1	3	–	–
^13^C leaves (mg l^−1^)	3	3	4.5	5	–	3	–

Replicates: 4 initials, 4 finals and 1 additional bottle for daily gas measurements (O_2_, CO_2_). Blank: water with complex microbial community, but without addition of daphnia or leaves; Daphnia: daphnia only; D:L: daphnia and leaves in a given ratio with a complex microbial community; Leaves: leaves only; b-DL1:1: daphnia and leaves (ratio 1:1) in bacterial community only.

Dissolved O_2_ content was measured daily in one bottle of each treatment (monitoring bottle) by inserting a microfibre optic oxygen sensor (Microx TX3, PreSense, Regensburg, Germany) through the septa. Subsequently, dissolved O_2,_^12^CO_2_ and ^13^CO_2_ were measured via a membrane inlet mass spectrometer (MIMS, Bay Instruments, Maryland, US). MIMS data were expressed as ratios to argon (Ar), as Ar is an inert gas that is not affected by any biochemical process. The daily O_2_, ^12^CO_2_ and ^13^CO_2_ measurements served as criteria to evaluate at which time point interactive effects would be most evident and to stop the incubations at that moment. The experiment was finalised after 11 incubation days. On the last incubation day, samples for isotopic analyses of dissolved CO_2_ were taken before sacrificing the bottles for final sampling. For analyses of particulate organic carbon (POC), PLFA and DNA, the bottles were shaken thoroughly and the whole content was filtered on muffled GF/75 filters (Advantec, 47-mm diameter, nominal pore size of 0.3 µm). The filters were then freeze-dried and subsampled for ^13^C, DNA and PLFA analyses. The flow-through was collected to measure dissolved organic carbon (DOC) and dissolved nitrogen with a TOC-L analyser (Shimadzu, Kyoto, Japan).

### Stable carbon isotope analyses and mixing model

Stable carbon isotopes were measured as gaseous (i.e., dissolved inorganic C) and particulate phase at the UC Davis Stable Isotope Facility. Water for dissolved CO_2_ analyses was sampled on the initial and final day, filled in 12-ml Labco Exetainer vials and fixed with ZnCl_2_. The samples were analysed for δ^13^C and ppm CO_2_ using a GasBench II system interfaced to a Delta V Plus isotope ratio mass spectrometer (Thermo Scientific, Bremen, Germany). Concentrations of CO_2_ in the water samples were calculated as described for headspace equilibration techniques by Oil and Halbedel [52].

For solid ^13^C analyses, freeze-dried GF/75 filters were encapsulated in tin capsules (EA Consumables, Inc.) and measured using an Elementar Vario EL Cube elemental analyser (Elementar Analysensysteme GmbH, Hanau, Germany) interfaced to a PDZ Europa 20–20 isotope ratio mass spectrometer (Sercon Ltd., Cheshire, UK). The daphnia carcasses and leaves used for the incubations were also sent for ^13^C measurement. The measuring accuracy is 0.2 per mill.

The formula for calculations of the direction and magnitude of non-additive interactive effects was adapted from Bengtsson et al. [21] as follows:

1, $${\mathrm{IE}} = \left( {\frac{{{\mathrm{CO}}_2({\mathrm{treatment}}) - {\mathrm{CO}}_2({\mathrm{controls}})}}{{{\mathrm{CO}}_2({\mathrm{controls}})}}} \right) \times 100$$

where IE refers to interactive effects (in %), CO_2_(treatment) is the CO_2_ production in the treatment of interest (i.e., where interactive effects are expected) and CO_2_(controls) is the sum of produced CO_2_ in the daphnia and leaf controls. For treatments DL1:3 and DL1:5 for which we did not have controls with the same amount of daphnia and leaves, CO_2_(controls) was calculated from CO_2_ production per mg daphnia and leaves.

To estimate the amount of daphnia- and leaf-derived POC and to trace the C source of the measured CO_2_, we used a two-source mixing model approach proposed by Cheng [53]. In treatments where daphnia and leaves were mixed, we calculated the CO_2_ fraction of the desired source as

2 $${\mathrm{f}}_{{\mathrm{source}}\;{\mathrm{A}}} = \frac{{({\updelta}^{{\mathrm{13}}}{\mathrm{CO}}_{2{\mathrm{sample}}}-{\updelta}^{{\mathrm{13}}}{\mathrm{C}}_{{\mathrm{source}}\;{\mathrm{B}}})}}{{({\updelta}^{{\mathrm{13}}}{\mathrm{C}}_{{\mathrm{source}}\;{\mathrm{A}}}-{\updelta}^{{\mathrm{13}}}{\mathrm{C}}_{{\mathrm{source}}\;{\mathrm{B}}})}}$$

When source A is *daphnia*, source B is *leaves* and vice versa. For daphnia- and leaf-derived POC, we used δ^13^C instead of δ^13^CO_2_ in Eq. 2. We did not apply the mixing model to the daphnia and leaf controls, but used total POC and CO_2_ concentrations as they contain only one C source. Based on Eq. 2, we calculated the normalised concentration of CO_2_ of the C source of interest as

3 $${\mathrm{CO}}_{2\, {\it{source}}\;{\mathrm{A}}} = \frac{{C_{{\mathrm{CO}}_2} \times f_{source\;A}}}{{dry\;weight_{source\;A}}}$$

A summary of the calculation steps for all samples is provided in Supplementary Table 1.

### Phospholipid-derived fatty acids analyses (PLFA)

Microbial cell enumeration can be difficult, particularly when considering partially particulate and colloidal OM sources such as daphnia carcasses. Previous studies demonstrated a clear suitability of PLFAs for this purpose [10, 54, 55]. Thus, we used PLFAs as an indicator for microbial biomass. Furthermore, PLFA can differentiate between major groups of organisms (e.g., prokaryotes vs. eukaryotes) at low taxonomic resolution [56]. Linking PLFAs with ^13^C analyses (i.e., compound-specific isotope analyses) reveals the C sources utilised by the different organisms to build up biomass. PLFAs were extracted from GF/75 filters for initial and final samples [57] and measured as fatty acid methyl esters (FAMEs) on a gas chromatographer (Agilent 6890, Germany) equipped with a silica capillary column (CP Sil 88 for FAME, Agilent) coupled to a mass-selective detector (Agilent 5973-N, Germany). FAMEs were identified by comparing retention times and mass spectra with a standard mixture of FAME (Bacterial Acid Methyl Esters Mixture, Matreya LLC, State College, US) and quantified using FAME-specific calibration curves. For the compound-specific isotope analyses, the four replicates were pooled due to the low concentrations of PLFAs in the samples. Individual PLFAs were analysed using a gas chromatographic system (Agilent 7890 Series GC) coupled via a combustion interface to a Finnigan MAT 253 isotope ratio mass spectrometer. Isotope ratios of individual biomarkers were expressed as ^13^C values in per mill relative to the V-PDB standard. The omega nomenclature was used to describe the detected FAMEs.

### DNA extraction and sequencing

Total DNA was extracted according to a modified protocol described by Nercessian et al. [58]. Briefly, filters and zirconia–silica beads were suspended in extraction buffer (CTAB), to which sodium dodecyl sulfate, lauroyl sarcosine and phenol–chloroform–isoamylalcohol were added before homogenising and centrifuging the samples. The aqueous phase was first transferred and washed with chloroform–isoamylalcohol and subsequently with polyethylene glycol. Nucleic acids were precipitated at 4 °C, washed with ethanol, air-dried and finally dissolved in ultra-pure water. The detailed protocol is available in the Supplementary Information. PCR, library preparation and sequencing was done by MrDNA (USA). The V3–V4 region of the 16S rRNA gene was amplified using bacterial primers 341F-785R [59], followed by library preparation (2 × 300 bp) and sequencing on a MiSeq (Illumina) platform. Fungal primers ITS4ngs (forward) and LF402 (reverse) were used for fungal community analysis [60].

### Statistical tests and bioinformatic approaches

Statistical significances and *t* values of O_2_, ^13^CO_2_ and PLFA concentrations were calculated using a two-way ANOVA test (Bonferonni’s multiple comparisons). Sequences were processed with the dada2 (ver. 3.6) R package [61] and taxonomy was assigned using the R package DECIPHER (ver. 2.16.1) [62]. Sequences were run against the SILVAv132 and LSUv11 database for identification of bacterial and fungal taxa. We tested for treatment-specific effects on both fungal and bacterial community composition using PERMANOVA following Hellinger transformation of the OTU matrix, using the Primer6 software package (Primer-E Ltd, Plymouth). Treatment-specific OTU enrichment was assessed using the multipatt function of the indicspecies (ver. 1.7.1) R package [63]. Specifically, relative OTU abundances were used to identify treatment-specific indicator species, allowing for multigroup comparisons. False discovery was minimised by applying a Bonferroni adjustment.

We used the WGCNA (ver. 1.68) R package for weighted correlation network analysis [64] to construct a topological overlap matrix (TOM) and test for the effects of external variables on the presence of different taxonomic groups. Briefly, rare OTUs (<0.01%) were excluded and samples were hierarchically clustered using the *hclust* function to identify outliers. We related external variables (such as daphnia or leaf-derived CO_2_ concentrations, O_2_, presence/absence of leaves or daphnia carcasses) to the samples and used this relationship as input for network construction and module detection. Network construction was performed using a soft-thresholding power of 6 and modules were detected using the TOM type *unsigned* and a minimum module size of 30. Modules were related to external variables by calculating module eigengenes and correlating those with the external variables. The results were visualised in a heatmap, where values of 1 correspond to a positive module-variable correlation and −1 to a negative correlation. The OTUs comprised within a module of interest were extracted and, thus, allowed to identify the microbial community related to specific variables.

## Results

**Fig. 1:**
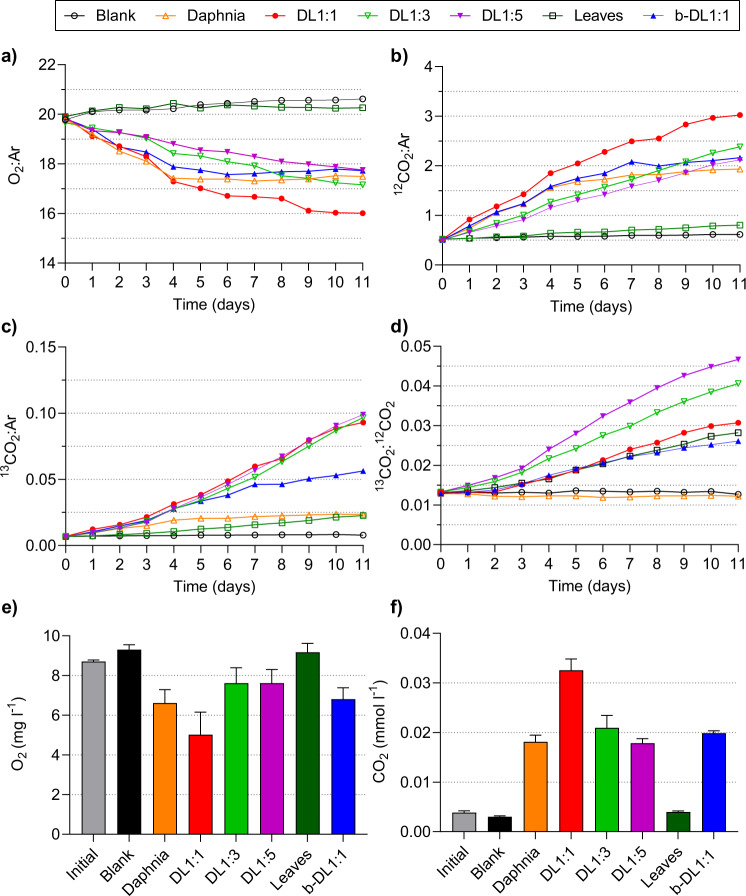
Microbial respiration across treatments during the incubation. Respiration measured daily in the monitoring bottles (not replicated) as ion currents (*m/z* ratios) and expressed as ratios of **a** O_2_ to Ar, **b** ^12^CO_2_ to Ar, **c** ^13^CO_2_ to Ar and **d** ^13^CO_2_ to ^12^CO_2_. Concentrations of O_2_ (**e**) and CO_2_ (**f**) measured in replicates (*n* = 4) at the beginning and at the end (day 11) of the experiment for each treatment. Blank: water with complex microbial community, but without addition of daphnia or leaves; Daphnia: daphnia only; D:L: daphnia and leaves in a given ratio with a complex microbial community; Leaves: leaves only; b-DL1:1: daphnia and leaves (ratio 1:1) in the bacterial community only.

Throughout 11 days of incubation, respiration rates of the microbial community, expressed either as O_2_ consumption or CO_2_ production, increased with increasing D:L ratios. The highest respiration rates were measured in the complex community treatment DL1:1, followed by treatments DL1:3 and DL1:5 (Fig. 1, Supplementary Fig. 1). O_2_ concentrations in the leaf controls were not significantly different from the blanks or the initials (Supplementary Table 2). In the daphnia control and bacterial treatment (b-DL1:1), respiration rates were high until day 4 but stagnated until the end of the incubation (Fig. 1a, b, Supplementary Fig. 1). The final O_2_ and CO_2_ concentrations measured on the last day in the replicated treatments (Fig. 1 e, f) supported the measurements in the daily monitoring bottles, i.e., the highest respiration in the complex community treatment DL1:1, comparable O_2_ and CO_2_ concentrations between the bacterial treatment (b-DL1:1) and the daphnia controls as well as between the blanks and the leaf controls. The initial C/N ratio of daphnia carcasses was 3.5 ± 0.15 (*n* = 5) and of the leaves 25.2 ± 2.2 (*n* = 5). DOC was (on purpose) very low and was completely consumed during the incubation (i.e., 0–0.5 mg l^−1^ in the finals, Supplementary Table 3). Hence, C was either incorporated into biomass (PLFAs) or respired (CO_2_).

**Fig. 2:**
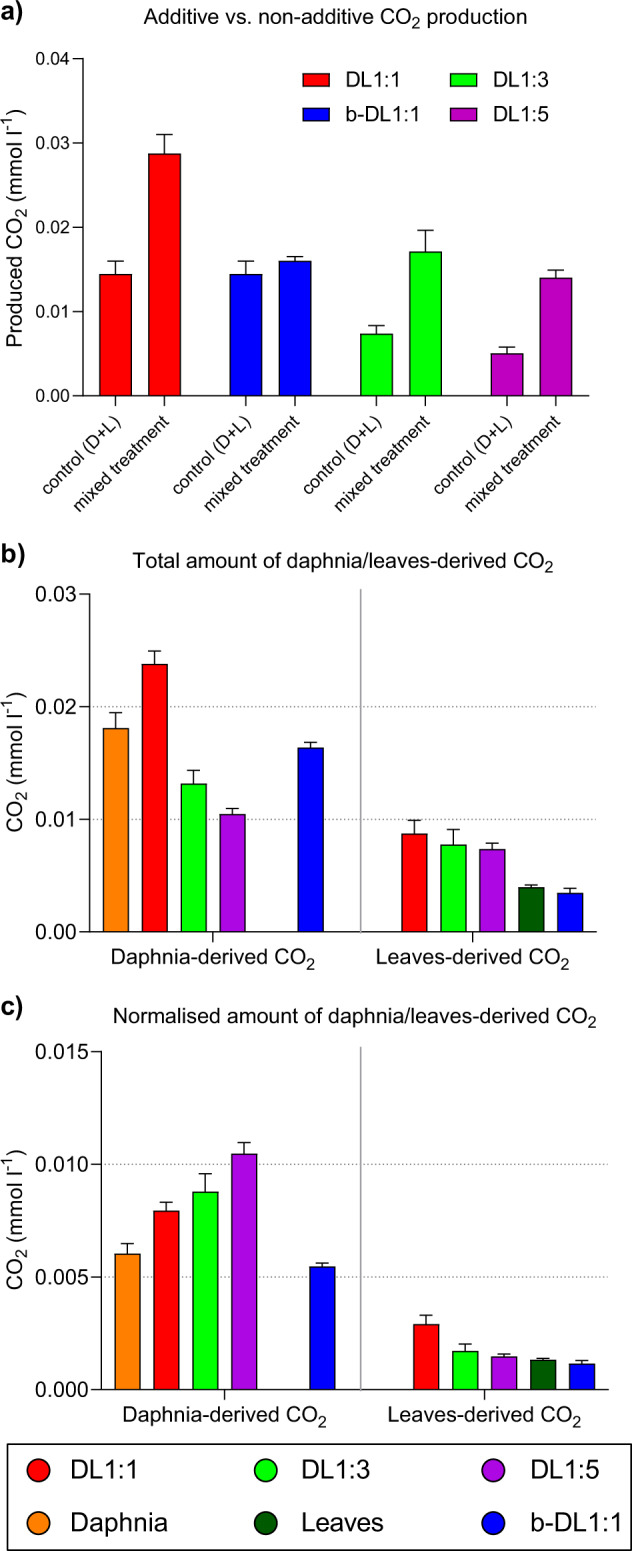
Interactive effects and source-specfic CO_2_ production. **a** Non-additive interactive effects measured as CO_2_ production in daphnia and leaves combined treatments compared to the sum of CO_2_ produced when daphnia and leaves were isolated (for DL1:3 and DL1:5, controls were calculated based on the CO_2_ production per mg daphnia or leaves in the controls for treatment DL1:1). **a** CO_2_ concentrations derived from the different carbon sources calculated with the two-source mixing model for the total amount of respired CO_2_ and **b** normalised by the amount of daphnia and/or leaf material in each treatment (*n* = 4). Daphnia: daphnia only; D:L: daphnia and leaves in a given ratio with a complex microbial community; Leaves: leaves only; b-DL1:1: daphnia and leaves (ratio 1:1) with a bacterial community only.

The turnover of POC increased with higher abundance of Daphnia carcasses, i.e., the final POC concentrations were the lowest in the daphnia-rich treatments (b-DL1:1, DL1:1) (for more information see the POC section of the Supplementary Information, Supplementary Fig. 2, Supplementary Tables 4 and 5). When comparing additive (i.e., sum of CO_2_ produced in separated daphnia and leaf controls) to non-additive CO_2_ production (i.e., the amount of CO_2_ produced in treatments where daphnia and leaves were mixed) in the DL1:1 treatment, we observed non-additive (interactive) effects of 98% (Fig. 2a). Because we only had controls for the DL1:1 treatment (i.e., 3 mg of leaves and 3 mg of daphnia in separated bottles), we calculated interactive effects normalised to the POC amounts as pseudocontrols for DL1:3 and DL1:5. The calculated interactive effect for DL1:3 was 131% and for DL1:5 178%. In the bacterial treatment (b-DL1:1), the non-additive effect accounted for 10% of CO_2_ production. However, since we only had controls with a complex microbial community, the magnitude of this effect might vary when comparing to controls with a reduced community complexity (bacterial community). CO_2_ derived from daphnia degradation was always significantly higher than leaf-derived CO_2_ of the same treatment (Fig. 2b, Supplementary Table 6). Absolute and normalised respiration rates from both daphnia- and leaf-derived CO_2_ were always significantly higher in the complex community treatment (DL1:1) compared to bacteria (b-DL1:1) (Fig. 2b, c, Supplementary Table 6). The highest absolute values of daphnia-derived CO_2_ were measured in the DL1:1 treatment (Fig. 2b). However, after normalising CO_2_ concentrations to the amount of daphnia added to the treatments, utilisation efficiency of daphnia-derived C increased with decreasing amounts of daphnia material in the treatments (i.e., the highest daphnia-derived CO_2_ concentrations in the DL1:5 treatment) (Fig. 2c). The highest leaf-derived CO_2_ concentrations per amount of leaves added were measured in the treatment with a high D:L ratio (i.e., DL1:1).

Some PLFAs have been commonly used as markers for fungal (e.g., 18:2ω6,9) or bacterial biomass (e.g., i15:0), while other unspecific PLFAs are generally used as universal biomarkers, covering prokaryotes and eukaryotes. By applying a cluster analysis to all PLFAs, we obtained two groups, which we assigned to eukaryotic and bacterial markers accordingly (Supplementary Fig. 3). The PLFAs 18:1ω9t and 18:1ω7c could not be separated chromatographically, thus they will be referred to as 18:1ω9t/7c further on. One cluster was composed of the PLFAs 18:1ω9t/7c and 18:2ω6,9, which are known to be present in daphnia [65] and fungal biomass [54]. Thus, these two PLFAs were used as indicators of eukaryotic biomass. The second cluster consisted mostly of bacteria-specific PLFAs (i15:0, a15:0, cy-17:0) and some unspecific PLFAs (16:1ω9, 18:1ω9c). Since the unspecific PLFAs clustered with bacterial-specific PLFAs and also showed a significant increase in the bacterial treatment, they were used as markers of bacterial biomass.

**Fig. 3:**
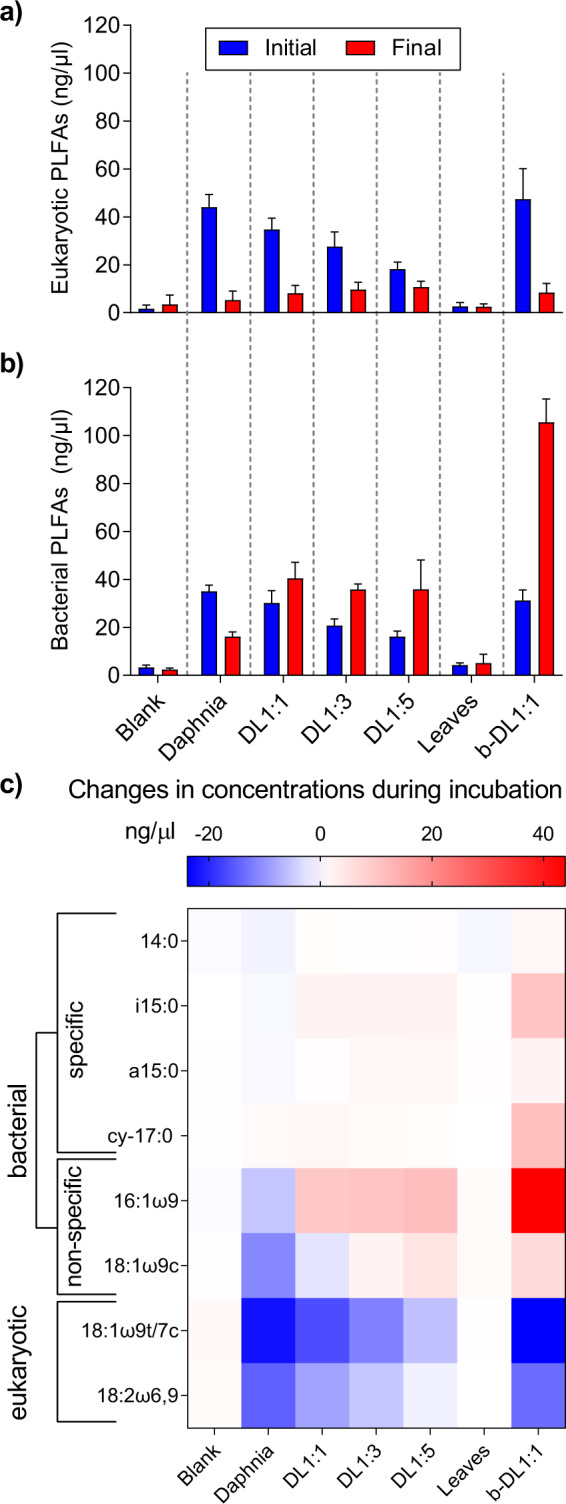
Changes in PLFA compositions during the incubation. Initial and final concentrations of **a** eukaryotic and **b** bacterial PLFAs; **c** concentration changes of specific PLFA across treatments during the incubation. Blank: water with complex microbial community, but without addition of daphnia or leaves; Daphnia: daphnia only; D:L: daphnia and leaves in a given ratio with a complex microbial community; Leaves: leaves only; b-DL1:1: daphnia and leaves (ratio 1:1) with a bacterial community only.

The initial samples revealed high abundances of eukaryotic PLFAs corresponding to the amendment of daphnia biomass to the treatments (Fig. 3a). Thus, the highest eukaryotic PLFA (i.e., mainly daphnia-derived PLFAs) concentrations were measured in the DL1:1 treatments (bacterial and complex microbial community) and the daphnia control (which corresponds to treatments where 3 mg of daphnia were added), but decreased with a lower D:L ratio. The final eukaryotic PLFA concentrations were not significantly different across treatments (daphnia, DL1:1, DL1:3, DL1:5 and b-DL1:1). When we consider the large contribution of daphnia carcasses to the eukaryotic PLFAs, the significantly lower final concentrations compared to the initial concentrations (Supplementary Table 7), indicate an efficient degradation of the initial daphnia biomass. Initial bacterial PLFA concentrations decreased with decreasing amounts of daphnia carcasses in the treatments (Fig. 3b), suggesting that the daphnia carcasses had an associated bacterial community. The final bacterial PLFA concentrations increased significantly in all DL treatments during the incubation period (Fig. 3b, Supplementary Table 7). A particularly steep increase of bacterial PLFA was observed in the bacteria-only treatment, which is mainly attributed to the enrichment of the bacteria-specific PLFAs i15:0 and cy-17:0 and the unspecific PLFAs 16:1ω9 and 18:1ω9 (Fig. 3c).

**Fig. 4:**
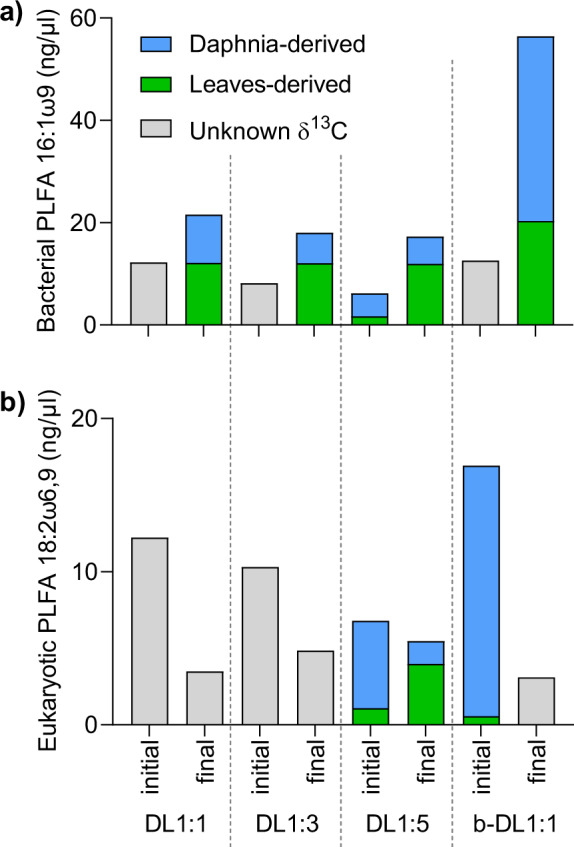
PLFA-specific isotope analyses. Amount of daphnia- and leaf-derived carbon of **a** bacterial PLFA 16:1ω9 and **b** eukaryotic PLFA 18:2ω6,9 based on the PLFA-specific ^13^C isotopic analysis. A two-source isotope-mixing model was applied to estimate for single PLFAs the proportion of carbon derived from either daphnia or leaves. Isotopic values could not be measured for every sample due to the low concentrations of PLFAs. Thus, grey bars indicate samples for which we have no ^13^C values. D:L: daphnia and leaves in a given ratio with a complex microbial community; b-DL1:1: daphnia and leaves (ratio 1:1) with a bacterial community only.

We generally observed a higher enrichment of the ^13^C label in the final samples compared to initials (Fig. 4). The amount of daphnia-derived C in two PLFAs (i.e., eukaryotic PLFA 18:2ω6,9 and bacterial PLFA 16:1ω9) decreased from initial values of 73–96% to 27–64% in the final samples (Supplementary Table 8). For these two PLFAs, the leaf-derived C increased from 3–27 % in the initials, to 35–72% in the finals.

### Microbial community composition

**Fig. 5:**
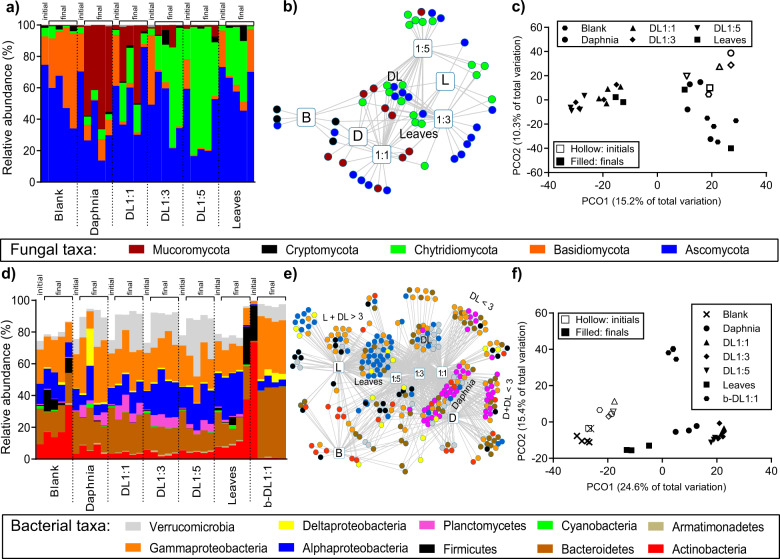
Microbial community analyses. Upper graphs: fungal taxonomic analyses: **a** relative abundances of taxa, **b** network analysis of indicator species and **c** PCO plots of fungal (ITS) sequences. Lower graphs: bacterial taxonomic analyses, **d** relative abundances of taxa. One sample from the daphnia controls and one from the leaf controls were identified as outliers (bar charts marked with a star) and excluded from the downstream analysis (i.e., network analysis, PCO plot); **e** network analysis of indicator species; **f** PCO plots of bacterial (16s) sequences. Blank: water with a complex microbial community, but without addition of daphnia or leaves; Daphnia: daphnia only; D:L: daphnia and leaves in a given ratio with a complex microbial community; Leaves: leaves only; b-DL1:1: daphnia and leaves (ratio 1:1) with a bacterial community only.

The fungal community composition in the roller bottles was initially dominated by Ascomycota and Basidiomycota. Addition of daphnia carcasses resulted in an increase in the relative abundance of Mucoromycota, particularly in the daphnia and DL1:1 treatments (Fig. 5a). Addition of leaf material resulted in an increase in the relative abundance of Chytridiomycota, an effect which was amplified in the DL1:3 and DL1:5 treatments (Fig. 5a). We found a significant difference amongst treatments (Fperm = 2.0123, *p* = 0.0001). More specifically, we found significant differences between treatments with high D:L ratios (1:1) and low DL ratios (1:5), or controls (Supplementary Table 9). Indicator species analyses identified only a few fungal indicators of individual treatments. We found Mucoromycota as indicators of high daphnia-to-leaf ratio treatments and Chytridiomycota as indicators of low daphnia-to-leaf ratio treatments (Fig. 5b). Treatments with daphnia and leaf addition formed a separate cluster (Fig. 5c).

The complex microbial community initially contained similar abundances of Actinobacteria, Bacteroidetes, Alphaproteobacteria and Gammaproteobacteria, whereas the bacteria-only treatment was dominated by small Actinobacteria and Firmicutes (Fig. 5d). With the addition of daphnia carcasses, we observed increases in the relative abundance of Bacteroidetes, Gammaproteobacteria, Planctomycetes and Verrucomicrobia in the complex community treatments (Fig. 5d). While Verrucomicrobia and a few Alphaproteobacteria emerged as indicators for the mixed treatments, Gammaproteobacteria were more likely to be indicators for low daphnia-to-leaf ratios and Planctomycetes for high daphnia-to-leaf ratios (Fig. 5e). Alphaproteobacteria increased in treatments containing leaf material (Fig. 5d), which was also reflected as strong indicator species for leaves (Fig. 5e). In the bacteria-only treatment (b-DL1:1), we saw a dominance of both Bacteroidetes and Gammaproteobacteria. More generally, the bacterial community composition was defined by the presence of the daphnia carcasses. Complex community treatments containing daphnia clustered together, while the bacteria-only treatment (b-DL1:1) formed a separate cluster (Fig. 5f). The community containing only leaf material did not differ substantially from the community where no POC source was provided (blank), or from initial samples (Fig. 5f).

**Fig. 6:**
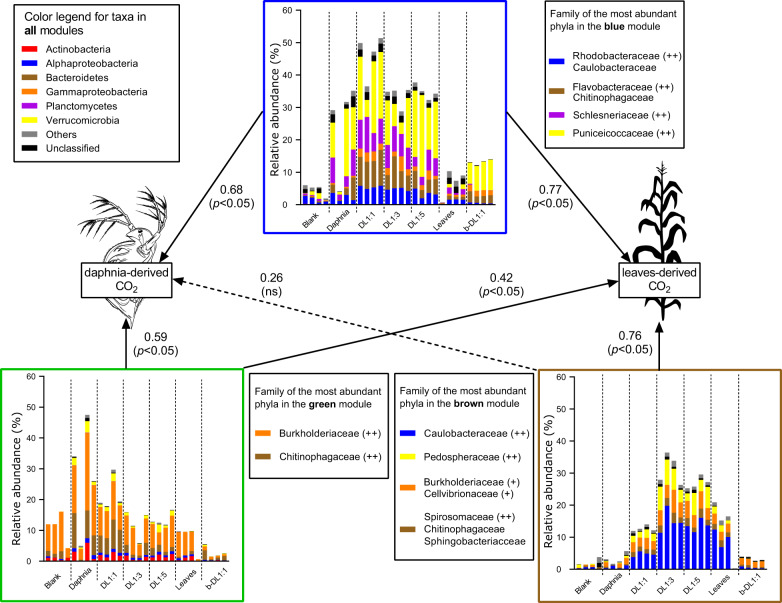
Relative abundance of bacterial taxa. Relative abundance of bacterial tax comprised within three selected WGCNA-based modules that were positively correlated with the external variables “daphnia-derived CO_2_” and/or “leaves-derived CO_2_”. External variables (i.e., CO_2_–) were used as input for network construction and module detection. The OTUs comprised within a module of interest were extracted and, thus, allowed to identify the microbial community related to specific variables. Arrows show the correlations between a module and the variables; continuous lines correspond to statistically significant correlations, dashed lines non-significant ones. Plus (+) symbols represent abundance of a certain family in a phylum (++: dominant family, +: abundant but not dominant, no sign: present but not abundant). Blank: water with a complex microbial community, but without addition of daphnia or leaves; Daphnia: daphnia only; D:L: daphnia and leaves in a given ratio with a complex microbial community; Leaves: leaves only; b-DL1:1: daphnia and leaves (ratio 1:1) with the bacterial community only.

Based on a WGCNA analysis of the bacterial 16S rRNA gene sequences, we explored modules that were positively correlated with the variables “daphnia-derived CO_2_” or “leaves-derived CO_2_” (Supplementary Fig. 4). Some modules comprised only few sequences or sequences that were dominant in single samples only (e.g., salmon module). We identified three modules that reflected best the bacterial communities linked to daphnia and/or leaf degradation (i.e., blue, brown and green modules, Fig. 6). Gammaproteobacteria (Burkholderiaceae) and Bacteroidetes (Chitinophagaceae) were common taxa in the green module, which was correlated to daphnia-derived CO_2_ (*ρ* = 0.59, *p* = 0.001) (Fig. 6). The brown module was correlated with leaf-derived respiration (*ρ* = 0.76, *p* = 3e^−6^) and was dominated by Alphaproteobacteria (exclusively Caulobacteraceae). Verrucomicrobia (Pedospheraceae), Gammaproteobacteria and some Bacteroidetes (mainly Spirosomaceae) were also present in this module, although at lower abundance (Fig. 6). The blue module was correlated with mixed treatments, i.e., leaf- (*ρ* = 0.77, *p* = 2e^−6^) and daphnia- (*ρ* = 0.68, *p* = 7e^−5^) derived CO_2_. The bacterial taxa within this module were predominantly composed of Verrucomicrobia (Puniceicoccaceae), Bacteroidetes (Flavobacteraceae), Alphaproteobacteria (Rhodobacteraceae) and Planctomycetes (Fig. 6).

## Discussion

Chemodiversity and microbial community complexity are a key component in OM degradation. We could show that the occurrence of zooplankton carcasses has the potential to stimulate the decomposition of leaf litter in aquatic environments. Furthermore, the magnitude of this enhanced decomposition appears to be also dependent on the degrading microbial community composition (i.e., bacteria only vs. bacteria and eukaryotic microorganisms).

### Interactions between OM sources and microbial activity

High respiration rates in treatments amended with daphnia (Fig. 1, Supplementary Fig. 1) and a very efficient utilisation of daphnia-derived OM (Fig. 2) supported our previous findings that daphnia carcasses are a bioavailable source of OM [37], which can easily be accessed and utilised by the microbial community. Trends in respiration were consistent with the microbial communities initially utilising OM derived from daphnia carcasses and progressively shifting their energy demands towards the less bioavailable leaf material.

Although slightly delayed, we observed a similar response to the daphnia controls in the simplified microbial community (b-DL1:1) in the presence of leaves (Fig. 1, Supplementary Fig. 1). This suggests that this simplified community was not as efficient in exploiting daphnia-derived OM as the complex community, or that whilst leaf-derived OM was available, it was not sufficient to sustain respiration beyond day 6–7. Higher ^13^C values of the very abundant bacterial PLFA 16:1ω9 (unspecific, Fig. 4), which contained 36% leaf-derived C (Supplementary Table 8), support a partial utilisation of the leaf-derived OM. If a small fraction of the soluble and bioavailable leaf compounds remained in the leaf material following leaching, the observed partial utilisation of leaf material could be attributed to the utilisation of leaf-derived (non-leached) compounds such as simple sugars. However, this would be inconsistent with the rapid leaching of the soluble fraction from leaf litter [51] and respiration rates in the leaf controls, which were only slightly higher than in the blanks. It is thus safe to assume a partial utilisation of leaf-derived lignin by the simplified community (b-DL1:1, Fig. 1).

### Interactions between eukaryotic microorganisms and bacteria in degrading OM

The removal of eukaryotes and large particles with their associated microbiota had manifold effects on the bacterial community structure and on OM cycling processes. These differences were expressed in the turnover rates of daphnia and leaf-derived C (Figs. 1, 2), levels of bacterial biomass (Fig. 3b) and in the bacterial community composition (Fig. 5d, f). Despite a much higher bacterial biomass in the bacteria-only treatment b-DL1:1 (Fig. 3b), the complex microbial community was more active (Figs. 1, 2). The initial community in the simplified community was less diverse than in the other treatments and was dominated by small bacteria (Actinobacteria, Firmicutes) (Fig. 5d). However, while the additional OM increased the diversity of the complex microbial community, the diversity of the simplified community remained low, instead dominated by copiotrophic Gammaproteobacteria and Bacteroidetes (Fig. 5d). Thus, the addition of the OM pools impacted the final microbial community as much, if not more, as the initial community composition. These differences indicate that eukaryotic microorganisms are important for shaping the bacterial community composition and sustaining a more diverse microbial community structure. We suggest two mechanisms that are responsible for this response: the presence of bacterial grazers is expected to control the growth of bacterial communities by predator-prey interactions, hindering the ability of single bacterial taxa to outcompete other groups and dominate the community [66]. This could partly explain the high bacterial PLFA concentrations (Fig. 3b) and the predominance of Gammaproteobacteria and Bacteroidetes (Fig. 5d) in the bacteria-only treatment, as these two phyla were associated with a high concentration of daphnia-derived OM (Fig. 5e). Consequently, the presence of daphnia-derived OM and the absence of bacterial predators could have led to an abundant copiotrophic bacterial community, lacking functional diversity and, therefore, with restricted capabilities to access the less bioavailable C of the leaf material [67, 68].

While bacteria might outcompete fungi in the fast consumption of easily accessible compounds (e.g., simple carbohydrates and amino acids) due to higher metabolic activities [9, 69], their ability to break down complex lignocellulosic compounds is more restricted [48]. Thus, bacteria are likely to become limited in energy and nutrients once most bioavailable resources have been used up. Saprotrophic fungi are known to play a decisive role in the decomposition of terrestrially derived OM [10, 40], as they can release extracellular enzymes or reactive oxygen species that break down or modify complex molecular structures like lignocellulosic compounds [70, 71]. This chemical transformation and degradation of large colloidal molecules releases smaller molecular products and by-products that, in turn, can become accessible to bacteria. Hence, even though fungi might be key organisms in breaking down the leaf material, bacteria might be as important when it comes to metabolising these degradation products [48]. Furthermore, increasing the relative abundances of Caulobacteraceae (Alphaproteobacteria) with increasing leaf content (Figs. 5d, 6) is consistent with the adaptation of Caulobacteraceae to low-nutrient conditions and their chemotactic capacity to trace and degrade lignocellulosic compounds even in very diluted environments [45, 72]. Both pathways would lead to an incorporation of leaf-derived C into bacterial biomass and explain the higher ^13^C enrichment in the bacterial PLFA 16:1ω9 in the complex microbial community treatments (56–69% leaf-derived C) compared to the bacterial treatment (35% leaf-derived C) (Fig. 4), highlighting the role of bacteria in processing (either directly or indirectly) leaf-derived C.

The fungal phylum Mucoromycota, which was enriched in daphnia controls, lacks laccases, required for the enzymatic breakdown of lignin derivatives, but exhibits high leucine aminopeptidase activity, and is therefore well suited for the breakdown of daphnia-derived OM [73]. Mucoromycota have also faster growth rates than Basidiomycota and Ascomycota [73], with their copiotrophic growth mirroring that of the Gammaproteobacteria and Bacteroidetes. Chytridiomycota, which are known to possess cellulose and hemicellulose-degrading enzymes [74, 75], were most abundant in the leaf-rich treatments (i.e., DL1:3, DL1:5 and leaf control) despite low initial abundances (Fig. 5a). Even though Ascomycota and Basidiomycota are prominent degraders of lignin derivatives [73, 74, 76], Basidiomycota were almost absent in the leaf-rich treatments and, while Ascomycota were still abundant, they generally decreased in relative abundances compared to the initials.

Experimental and methodological restrictions limit our capabilities to clearly disentangle single pathways of energy and nutrient flows from microbial cross-feeding or other feedback processes. Higher bacterial abundances, lower diversity and microbial activity in the bacteria-only treatment could be equally explained by the absence of bacterial grazers, leading to a low bacterial and functional diversity in regard to OM utilisation [67, 68], as by the absence of fungi or lignocellulose-degrading bacteria, which could initiate the degradation of complex compounds and trigger cascading processes of OM turnover. Therefore, we cannot evaluate the role of specific processes or interactions, since each of them seems equally important to the biogeochemical function. Equally, the relative impact of these processes throughout the year, as bacterial, fungal and grazing abundances fluctuate, would likely vary and have impacts on turnover of both OM pools. Yet, this study provides evidence for close and complex links between the different levels of microbial interactions, chemodiversity and OM cycling processes.

### Interactive effects induced by daphnia carcasses and leaf OM

The combined presence of both daphnia carcasses and leaves resulted in increased microbial respiration and a more efficient turnover of both OM sources, than we observed for each individual OM source (Fig. 2). While this effect almost doubled (increase of 98%) in the complex community treatment DL1:1, it only increased by 10% in the bacterial community b-DL1:1 compared to the controls. However, we only had controls with a complex microbial community, and thus interactive effects of the bacterial community are likely to be higher than 10% when comparing to bacterial controls. This implies that twice as much CO_2_ was produced by the complex community with both OM sources combined, than was when both OM sources were degraded in isolation (Fig. 2a). Even though in absolute terms non-additive CO_2_ production was the highest in the DL1:1 treatment, in relative terms, non-additive interactive effects were the highest in the low daphnia-to-leaf ratios (130% in DL1:3, 178% in DL1:5), indicating a more efficient utilisation when bioavailable daphnia-derived OM is limiting. This was also reflected in higher daphnia-derived CO_2_ concentrations (normalised) with decreasing abundances of daphnia carcasses in the treatments (Fig. 2c). These findings are consistent with the presence of a positive non-additive effect in aquatic ecosystems, similar to that observed in terrestrial ecosystems [19, 20]. Previous studies addressing this topic either supported [26, 27, 29] or refuted [77, 78] the presence of non-additive interactive effects in an experimental setup. A substantial part of the discrepancy in the results stems from differences in experimental and methodological approaches [21]. The approaches differed mainly in the microbial inoculum (single bacterial isolates, bacterial communities, fungal isolates, mixed or natural microbial communities), in the C substrates used (pure or synthetic C compounds vs. different natural OM substrates) and in the response variables used (CO_2_, DOC, POC and bacterial and/or fungal growth). By combining a wide array of methodological approaches to different experimental setups (chemodiversity vs. community complexity), we were able to identify some key factors and processes affecting non-additive interactive effects. We have shown that the responses differed between complex microbial and bacterial communities only, and that using a different community would lead to other or variable results, even in the same experimental setup (Fig. 1). Furthermore, only few studies have addressed the effects of different fractions of bioavailable OM for the degradation rates of more persistent OM pools [9, 77]. Whilst the highest respiration rates of leaf-derived material were measured in the treatment with the highest abundances of daphnia carcasses (i.e., DL1:1), the presence of leaves also stimulated the degradation of daphnia-derived OM (Fig. 2c, d), indicating that chemodiversity and the ratio of bioavailable to more persistent OM sources is an important factor that needs to be considered in studies targeting processes involved in OM turnover. We strongly encourage future studies, aiming to further explore the underlying mechanisms of non-additive interactive effects, to combine biogeochemical approaches (e.g., double-source labelling: ^13^C and ^15^N) to innovative analytical techniques (e.g., nanoSIMS, CARD-FISH and DNA-SIP) and bioinformatics (e.g., metagenomics, transcriptomics and proteomics). Such interdisciplinary approaches have the potential to clearly link OM utilisation to specific taxa and community responses via gene expression and functional enzymes amongst others.

**Fig. 7:**
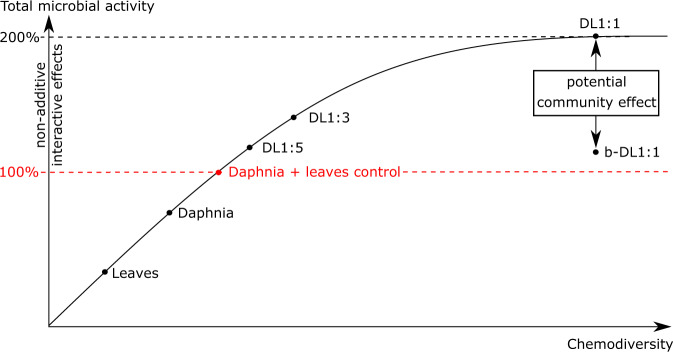
Total microbial activity and non-additive interactive effects as a function of organic matter chemodiversity. Non-additive interactive effects start above the red line, which visualises the sum of microbial activity (i.e., CO_2_ production) when carbon sources are isolated. In the zone of non-additive interactive effects, the increase of microbial activity is not linear and reaches a steady state when chemodiversity is high. Furthermore, we suggest a community effect that influences the slope and the maxima of the curve: while DL1:1 and b-DL1:1 had the same conditions in regard to organic matter chemodiversity, DL1:1 had the highest microbial activity resulting from a high community complexity, compared to b-DL1:1- simplified bacterial community composition. Daphnia: daphnia only; D:L: daphnia and leaves in a given ratio with a complex microbial community; Leaves: leaves only; b-DL1:1: daphnia and leaves (ratio 1:1) with the bacterial community only.

The more bioavailable and chemically diverse compounds (e.g., proteins, lipids and chitin from zooplankton carcasses) are available to provide the degrading community with sufficient energy and diverse nutrients, the more it will allow a complex microbial community to exploit C from additional persistent OM sources to sustain growth. While leaves were relatively poor in nutrients (C/N = 25), they provided abundant C to complement the nutrient-rich, daphnia-derived OM (C/N = 3.5) and maintain a stoichiometric balance for microbial growth. Based on these results, we conclude that non-additive interactive effects are a function of OM chemodiversity and microbial community complexity (Fig. 7). This is consistent with a previous study by Tanentzap et al. [79], addressing the relationship between chemodiversity and microbial diversity on the turnover rates of OM in lakes. Our experimental approach revealed that if OM sources do not provide enough biochemical diversity (e.g., leaves and daphnia controls), even a complex microbial community will face C or nutrient limitations. On the other hand, a system with high chemodiversity but a low microbial community complexity (e.g., bacterial treatment, b-DL1:1), is also restricted in growth as a result of limited access to C and nutrients due to lacking functional diversity. While chemodiversity is expected to have a stronger effect on the microbial complexity than vice versa, the degrading microbial community also has an impact on the complexity of the surrounding OM pool [79, 80]. Consequently, only a system combining both, a complex microbial community and a chemically diverse OM pool, is likely to sustain microbial growth and maximise net OM turnover rates.

Freshwater environments worldwide are facing increasing C and nutrient loads, changes in phytoplankton compositions [81] and declining zooplankton abundances [82, 83]. The tight interaction between chemical and microbial diversity, highlighted in this study and elsewhere, should be considered in the context of overall C turnover in aquatic ecosystems, since long-term changes and seasonal pulses of allochthonous and autochthonous OM input can affect both aquatic chemodiversity and microbial diversity, impacting net remineralisation rates in response [79]. Zooplankton carcasses are an important and up to now largely neglected piece of the puzzle with regard to OM degradation in marine and freshwater ecosystems, and should be considered in more detail in future studies of C cycling.

## Supplementary information

Supplementary information
