# Supplementary material for: Zooplankton carcasses stimulate microbial turnover of allochthonous particulate organic matter
Source: ISME J. 2021 Jan 18;15(6):1735–50. doi: 10.1038/s41396-020-00883-w (PMC8163850; doi:10.1038/s41396-020-00883-w)
Supplement: Supplementary file 1 — Supplementary information [file 41396_2020_883_MOESM1_ESM.docx]

**Supplementary information**

**Preparation of inocula**

For the preparation of the inoculum, 20 litres of surface water were sampled in April 2018 from the shore, pre-filtered on a 50 µm zooplankton mesh and centrifuged at low velocity (500 RCF, 10 min) to collect organisms sensible to strong centrifugation (e.g. fungi, grazers) without damage. Ten ml of water were collected from the bottom of the centrifugation tube serving as eukaryotic inoculum (which would also include some bacteria). The remaining supernatant was centrifuged at high velocity (10000 RCF, 10 min) to concentrate the bacterial community into pellets. Both, eukaryotic and bacterial concentrates, were mixed to obtain a concentrated inoculum of a complex microbial community (including prokaryotes and eukaryotes <50 µm). Additionally, 1 ml of pre-filtered natural lake water was added to the complex microbial community treatments in case that the centrifugation procedure might have excluded some organisms in the inoculum. One treatment (b-DL1:1, see **Table 1**) was solely inoculated with a bacterial community. For this treatment lake water was filtered on 0.8 µm filters and the flow-through was centrifuged at 10000 RCF for 10 minutes to concentrate the bacterial community < 0.8 µm. The natural bacterial concentration of lake Fuchskuhle is in the order of 5*10^7^ cells*ml^-1^. If we assume that the centrifugation process concentrates the bacterial community by a factor of 50 (we centrifuge 500 ml lake water and collected 10 ml from the bottom) we end up with a total concentration of 2.5*10^9^ cells*ml^-1^ in the inoculum. Thus, by adding 1 ml of inoculum to 1 l we reach an initial bacterial concentration roughly in the range of 2.5*10^6^ cells*ml^-1^. In the bacterial treatment we filtered the lake water on 0.8 µm prior to the centrifugation. Thus we might have reduced the initial bacterial density by half to approximately 2*10^7^ cells*ml^-1^ (prior to centrifugation). If we calculate the concentration factor as above (x50) and a dilution factor of 1000 (1 ml inoculum in 1l) we end up with an estimation of the initial bacterial density of roughly 10^6^ bacterial cells in the initial bacterial treatment. Thus, an initial bacterial density of 10^6^ would be a bit lower, but still in the range of natural bacterial densities.

**DNA extraction**

Zirconium and glass beads of various diameter and 0.6 mL CTAB (cetyltrimethyl-ammonium bromide) buffer were added to the samples. Then 60 µl of 10% sodium dodecyl sulfate (w:v), 60 µl of 10% N-Lauroylsarcosin (w:v), and 0.6 mL of pH-neutral phenol-chloroform-isoamylalcohol mixture (25:24:1, v:v:v) were added. Samples were homogenized on a vortexer for 10 min at highest speed and then centrifuged at 16000×g for 10 min at 4 °C. The aqueous phase was transferred into new reaction tubes, washed with 1 volume of chloroform-isoamylalcohol (24:1, v:v) and centrifuged at 16000×g for 10 min at 4 °C. Again, the aqueous phase was transferred into new reaction tubes and mixed with 2 volumes of 30% polyethylene glycol (w:v) in 1.6 M NaCl. After incubation for 1.5 h at 4 °C samples were centrifuged at 17000× g for 60 min at 4 °C. The supernatant was removed and the pellet was washed with 1 mL of ice-cold 70% ethanol. After centrifugation at 17000×g for 10 min the supernatant was removed and the nucleic acid pellet was air-dried and finally dissolved in 50 µL ultra-pure water.

**Supplementary table 1:** Detail steps of the mixing model calculations to obtain total and normalized CO_2_ concentrations derived from either daphnia or leaves, shown in **Figure 2**. **b-DL1:1**: daphnia and leaves (ratio 1:1) with bacterial community only; **D:L**: daphnia and leaves in a given ratio with a complex microbial community.

|  | A | B | C | D | E | F | G | H |
| --- | --- | --- | --- | --- | --- | --- | --- | --- |
|  |  |  | fraction derived from daphnia or leaves: | | total respiration: | | normalized respiration: | |
| Sample | CO_2_ (mmol l^-1^) | δ^13^C VPDB | f_daphnia_ | f_leaves_ | daphnia CO_2_ (mmol l^-1^) | leaves CO_2_ (mmol l^-1^) | daphnia CO_2_ (mmol l^-1^) | leaves CO_2_ (mmol l^-1^) |
| calculation: |  |  | =(B-δ^13^C_leaves_)/(δ^13^C_daphnia_ - δ^13^C_leaves_) | =(B-δ^13^C_daphnia_)/(δ^13^C_leaves_ - δ^13^C_daphnia_) | = A*C | = A*D | =E/mg_daphn._ | =F/mg_leaves_ |
| b-DL1:1 | 0,0206 | 1246 | 0,81 | 0,19 | 0,0167 | 0,0038 | 0,0056 | 0,0013 |
| b-DL1:1 | 0,0197 | 1177 | 0,82 | 0,18 | 0,0162 | 0,0035 | 0,0054 | 0,0012 |
| b-DL1:1 | 0,0195 | 1248 | 0,81 | 0,19 | 0,0158 | 0,0037 | 0,0053 | 0,0012 |
| b-DL1:1 | 0,0197 | 978 | 0,85 | 0,15 | 0,0168 | 0,0029 | 0,0056 | 0,0010 |
| DL1:1 | 0,0340 | 1903 | 0,72 | 0,28 | 0,0243 | 0,0097 | 0,0081 | 0,0032 |
| DL1:1 | 0,0307 | 1659 | 0,75 | 0,25 | 0,0231 | 0,0076 | 0,0077 | 0,0025 |
| DL1:1 | 0,0350 | 1885 | 0,72 | 0,28 | 0,0252 | 0,0099 | 0,0084 | 0,0033 |
| DL1:1 | 0,0306 | 1722 | 0,74 | 0,26 | 0,0227 | 0,0079 | 0,0076 | 0,0026 |
| DL1:3 | 0,0219 | 2582 | 0,62 | 0,38 | 0,0135 | 0,0084 | 0,0090 | 0,0019 |
| DL1:3 | 0,0234 | 2558 | 0,62 | 0,38 | 0,0145 | 0,0089 | 0,0097 | 0,0020 |
| DL1:3 | 0,0175 | 2242 | 0,67 | 0,33 | 0,0117 | 0,0058 | 0,0078 | 0,0013 |
| DL1:3 | 0,0210 | 2520 | 0,62 | 0,38 | 0,0131 | 0,0079 | 0,0087 | 0,0017 |
| DL1:5 | 0,0169 | 2744 | 0,59 | 0,41 | 0,0100 | 0,0069 | 0,0100 | 0,0014 |
| DL1:5 | 0,0184 | 2908 | 0,57 | 0,43 | 0,0104 | 0,0080 | 0,0104 | 0,0016 |
| DL1:5 | 0,0172 | 2712 | 0,60 | 0,40 | 0,0103 | 0,0070 | 0,0103 | 0,0014 |
| DL1:5 | 0,0188 | 2734 | 0,59 | 0,41 | 0,0112 | 0,0076 | 0,0112 | 0,0015 |
| δ^13^C values of sources: | | δ^13^C_daphnia_ = -21.29; δ^13^C_leaves_ = 6753 | | |  |  |  |  |


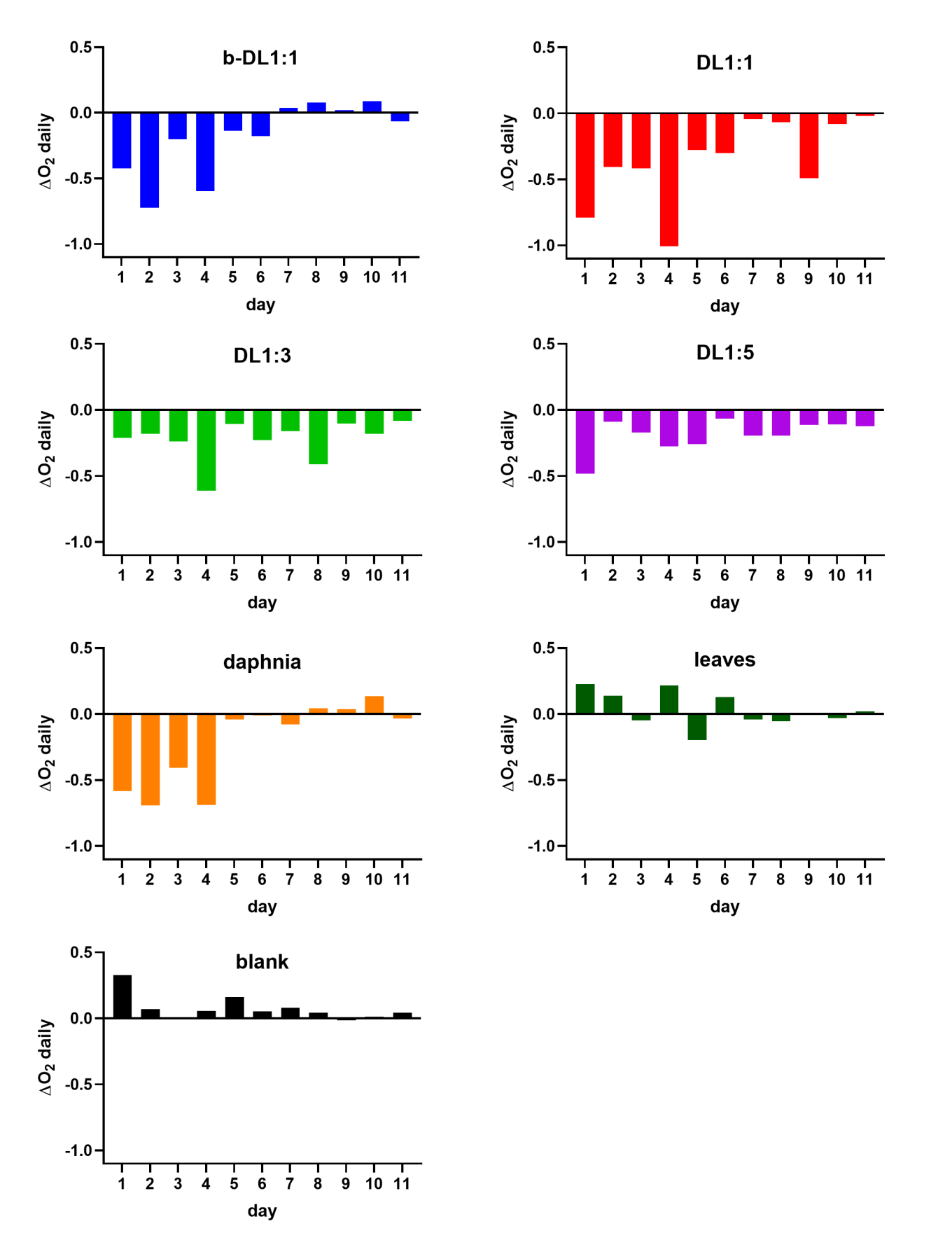


**Supplementary figure 1:** Daily respiration rates calculated as daily O_2_ consumption based on the daily MIMS measurements (m/z ratios). ΔO_2_ are relative values and not absolute O_2_ concentrations. **b-DL1:1**: daphnia and leaves (ratio 1:1) with bacterial community only; **D:L**: daphnia and leaves in a given ratio with a complex microbial community; **Daphnia:** daphnia only; **Leaves:** leaves only; **Blank**: microbial community without any OM addition.

**Supplementary table 2:** Summary of a one-way ANOVA test (Tukey’s multiple comparisons) of the O_2_ concentrations measured in the initials and final treatments (**Figure 1**). Numbers correspond to the adjusted p-values and statistical significances are expressed as cell shade (light grey = significant; dark grey = not significant).

|  | **blank** | **daphnia** | **DL1:1** | **DL1:3** | **DL1:5** | **leaves** | **b-DL1:1** |
| --- | --- | --- | --- | --- | --- | --- | --- |
| **initials** | 0.9 | 0.003 | <0.0001 | 0.4 | 0.3 | >0.9 | 0.008 |
| **blank** |  | 0.0001 | <0.0001 | 0.04 | 0.02 | >0.9 | 0.0004 |
| **daphnia** |  |  | 0.03 | 0.5 | 0.4 | 0.0003 | >0.9 |
| **DL1:1** |  |  |  | 0.0006 | 0.0002 | <0.0001 | 0.01 |
| **DL1:3** |  |  |  |  | >0.9 | 0.07 | 0.7 |
| **DL1:5** |  |  |  |  |  | 0.04 | 0.7 |
| **leaves** |  |  |  |  |  |  | 0.0007 |

**Supplementary table 3:** Dissolved organic carbon (DOC) and dissolved nitrogen (DN) measured as mg l^-1^. DOC and DN concentrations were overall very low, as no additional source of dissolved OM was added besides what leached from daphnia carcasses upon addition. Values in **bold** were below the level of quantification, i.e. < 0,15 mg l^-1^ for DOC and <0,1 mg l^-1^ for DN. **Blank**: water with a complex microbial community, but without addition of daphnia or leaves: **Daphnia:** daphnia only; **D:L**: daphnia and leaves in a given ratio with a complex microbial community; **Leaves:** leaves only; **b-DL1:1**: daphnia and leaves (ratio 1:1) with bacterial community only.

|  | DOC (mg l^-1^) | | DN (mg l^-1^) | | DOC/DN | |
| --- | --- | --- | --- | --- | --- | --- |
| **treatment** | **initial** | **final** | **initial** | **final** | **initial** | **final** |
| **b-DL1:1** | 0,33 | **<0,15** | 0,40 | 0,50 | 0,83 | NA |
|  | 0,30 | **<0,15** | 0,40 | 0,50 | 0,75 | NA |
|  | 0,32 | **<0,15** | 0,40 | 0,50 | 0,80 | NA |
|  | 0,29 | **<0,15** | 0,40 | 0,50 | 0,73 | NA |
| **DL1:1** | 0,34 | 0,26 | 0,40 | 0,50 | 0,85 | 0,52 |
|  | 0,31 | **<0,15** | 0,40 | 0,50 | 0,78 | NA |
|  | 0,27 | **<0,15** | 0,40 | 0,50 | 0,68 | NA |
|  | 0,35 | **<0,15** | 0,40 | 0,50 | 0,88 | NA |
| **DL1:3** | 0,08 | 0,29 | 0,40 | 0,40 | 0,20 | 0,73 |
|  | 0,15 | **<0,15** | 0,40 | 0,40 | 0,38 | NA |
|  | **<0,15** | **<0,15** | 0,40 | 0,40 | NA | NA |
|  | **<0,15** | **<0,15** | 0,40 | 0,40 | NA | NA |
| **DL1:5** | **<0,15** | 0,36 | 0,40 | **<0,1** | NA | NA |
|  | **<0,15** | 0,37 | 0,40 | 0,05 | NA | 7,40 |
|  | **<0,15** | 0,36 | 0,40 | 0,05 | NA | 7,20 |
|  | 0,16 | 0,37 | 0,40 | 0,06 | 0,40 | 6,17 |
| **daphnia** | 0,38 | 0,53 | 0,23 | **<0,1** | 1,65 | NA |
|  | 0,39 | 0,52 | 0,23 | **<0,1** | 1,70 | NA |
|  | 0,40 | 0,48 | 0,18 | **<0,1** | 2,22 | NA |
|  | 0,39 | 0,51 | 0,22 | **<0,1** | 1,77 | NA |
| **leaves** | 0,33 | 0,35 | **<0,1** | **<0,1** | NA | NA |
|  | 0,35 | 0,34 | **<0,1** | **<0,1** | NA | NA |
|  | 0,37 | **<0,15** | **<0,1** | 0,19 | NA | NA |
|  | 0,34 | 0,34 | **<0,1** | **<0,1** | NA | NA |
| **blank** | 0,33 | 0,37 | **<0,1** | **<0,1** | NA | NA |
|  | 0,34 | 0,40 | **<0,1** | **<0,1** | NA | NA |
|  | 0,37 | 0,34 | **<0,1** | **<0,1** | NA | NA |
|  | 0,35 | 0,34 | **<0,1** | **<0,1** | NA | NA |

**POC (particulate organic carbon)**

The daphnia carcasses had a C/N ratio of 3.5 ± 0.15 and the C/N ratio of the leaves was 25.2 ± 2.2. In both treatments with a daphnia- to leaves-derived OM ratio of 1:1 (i.e. DL1:1 and b-DL1:1) POC was significantly lower at the end of the incubation compared to initial values (Supplementary figure 2a). While not statistically significant, we did also observe lower POC in the finals of treatment DL1:3, DL1:5 and the daphnia controls. The leaves controls contained a non-significantly higher POC content at the end of the incubation (Figure 2a), indicative of a microbial colonisation, but not utilisation (Supplementary table 2). The two-source mixing model estimations of the POC origin reflected the daphnia- to leaves-derived OM ratios in the initial samples (Supplementary figure 2b). Daphnia and leaves-derived POC was always lower at the end of the incubation compared to initial values, however, these differences were never statistically significant (Supplementary table 3). This might be due to the large variance of the initial POC values, which we attribute to methodological reasons (daphnia carcasses and leaves of the initial samples were not degraded yet and distributed more heterogeneously on the filter compared to final samples). ***
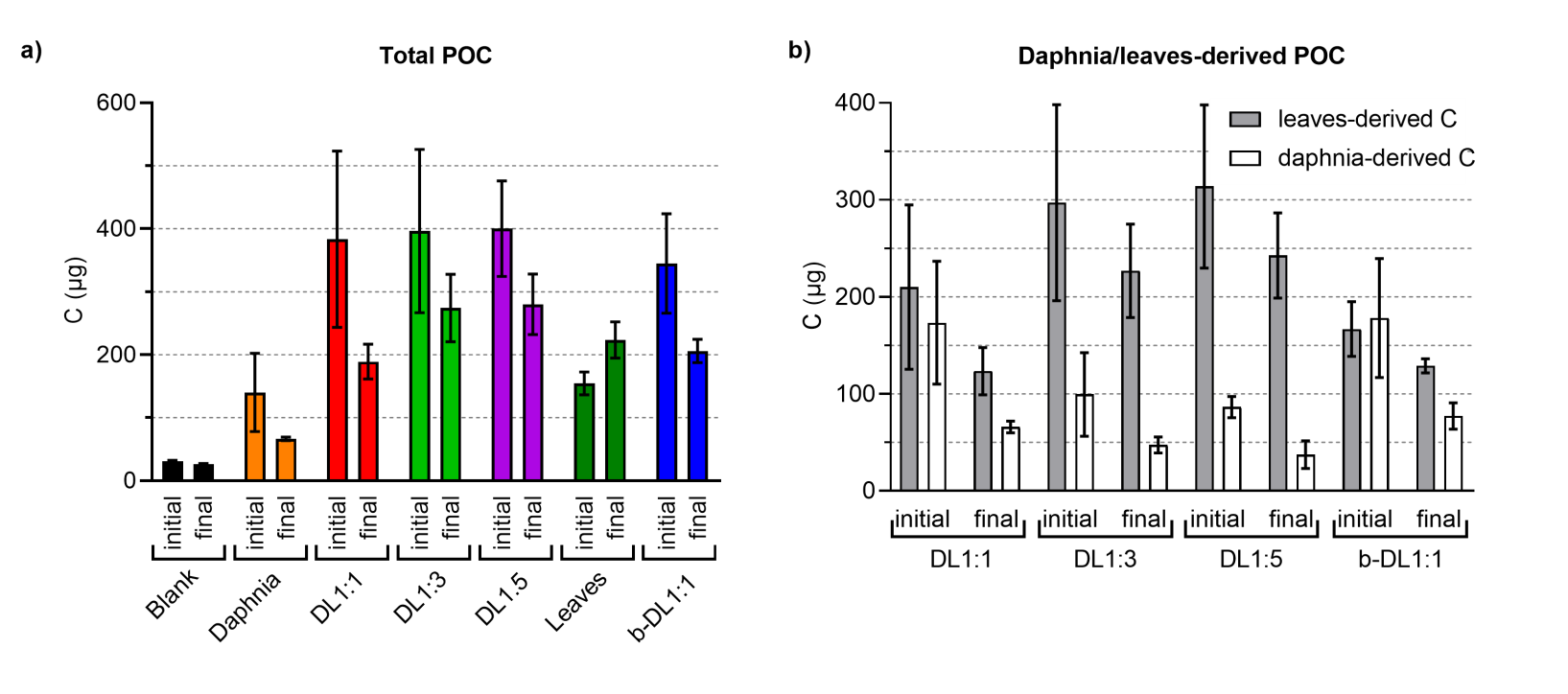
***

**Supplementary figure 2:** **a)** Total particulate organic carbon (POC) and **b)** daphnia and leaves-derived POC calculated using an isotopic mixing model approach. Note: the mixing model approach of POC does not allow differentiating between non-degraded source-OM and microbially processed/transformed OM (e.g. non-degraded leaves and biomass of microbes which consumed leaf material will have similar ^13^C isotopic values and will both be attributed to “leaves-derived C”. **b-DL1:1**: daphnia and leaves (ratio 1:1) with bacterial community only; **D:L**: daphnia and leaves in a given ratio with a complex microbial community; **Daphnia**: daphnia only; **Leaves**: leaves only; **Blank**: microbial community without any OM addition.

**Supplementary table 4:** Summary of two-way ANOVA test (Bonferonni’s multiple comparisons) of the daphnia and leaves-derived POC concentrations calculated based on a two-source mixing model (**Supplementary figure 2b**). Numbers correspond to the t-values and statistical significances are expressed as cell shade (light grey = significant; dark grey = not significant).

| Total POC | | | | | | | |
| --- | --- | --- | --- | --- | --- | --- | --- |
| Statistical differences between initial and final POC within same treatment | | | | | | | |
| Blank | Daphnia | | DL1:1 | DL1:3 | DL1:5 | Leaves | b-DL1:1 |
| 0.1089 | 1.675 | | 4.079 | 2.772 | 2.726 | 1.562 | 3.152 |
| Statistical differences of final POC across treatments | | | | | | | |
|  | Daphnia | | DL1:1 | DL1:3 | DL1:5 | Leaves | b-DL1:1 |
| Blank | 0.91 | | 3.692 | 5.621 | 5.752 | 4.469 | 4.071 |
| Daphnia |  | | 2.782 | 4.711 | 4.842 | 3.559 | 3.161 |
| DL1:1 |  | |  | 1.929 | 2.06 | 0.777 | 0.379 |
| DL1:3 |  | |  |  | 0.1315 | 1.152 | 1.55 |
| DL1:5 |  | |  |  |  | 1.283 | 1.681 |
| Leaves |  | |  |  |  |  | 0.398 |
|  |  |  | p-value <0.05 | |  |  |  |
|  |  |  | p-value >0.05 | |  |  |  |

**Supplementary table 5:** Summary of two-way ANOVA test (Bonferonni’s multiple comparisons) of the daphnia and leaves-derived POC based on a two-source mixing model (**Supplementary figure 2b**).

| Statistical differences of POC mixing model values: initial vs. final | | | | | | | | | | | | |
| --- | --- | --- | --- | --- | --- | --- | --- | --- | --- | --- | --- | --- |
|  | Daphnia-derived | | | | | | Leaves-derived | | | | | |
|  | Significant? | | | t value | | | Significant? | | | t value | | |
| DL1:1 | Yes | | | 4.133 | | | No | | | 1.886 | | |
| DL1:3 | No | | | 2.165 | | | No | | | 1.646 | | |
| DL1:5 | No | | | 2.036 | | | No | | | 1.671 | | |
| b-DL1:1 | Yes | | | 4.191 | | | No | | | 0.8927 | | |
| Statistical differences of POC mixing model values across treatments | | | | | | | | | | | | |
|  | | Initial values | | | | | | final values | | | | |
|  | | daphnia-derived | | | leaves-derived | | | daphnia-derived | | | leaves-derived | |
|  | | signific. | t value | | signific. | t value | | signific. | t value | | signific. | t value |
| DL1:1 vs. DL1:3 | | No | 2.841 | | No | 1.891 | | No | 0.7701 | | No | 2.434 |
| DL1:1 vs. DL1:5 | | Yes | 3.342 | | No | 2.257 | | No | 1.183 | | No | 2.804 |
| DL1:1 vs. b-DL1:1 | | No | 0.1833 | | No | 0.9427 | | No | 0.4711 | | No | 0.1265 |
| DL1:3 vs. DL1:5 | | No | 0.5418 | | No | 0.3950 | | No | 0.4124 | | No | 0.3695 |
| DL1:3 vs. b-DL1:1 | | Yes | 3.266 | | Yes | 3.061 | | No | 1.241 | | No | 2.308 |
| DL1:5 vs. b-DL1:1 | | Yes | 3.808 | | Yes | 3.456 | | No | 1.654 | | No | 2.677 |

**Supplementary table 6:** Summary of two-way ANOVA test (Bonferonni’s multiple comparisons) of the daphnia and leaves-derived CO_2_ concentrations calculated based on a two-source mixing model (**Figure 2 c,d**). Numbers correspond to the t-values and statistical significances are expressed as cell shade (light grey = significant; dark grey = not significant).

| Statistical differences of daphnia vs. leaves-derived CO_2_ within the same treatment | | | | | | | | | | | | | | | | | |
| --- | --- | --- | --- | --- | --- | --- | --- | --- | --- | --- | --- | --- | --- | --- | --- | --- | --- |
| DL1:1 | | | | DL1:3 | | | | | DL1:5 | | | | | b-DL1:1 | | | |
| 25.18 | | | | 9.062 | | | | | 5.191 | | | | | 21.58 | | | |
| Total amount of respired CO_2_ | | | | | | | | | | | | | | | | | |
| daphnia-derived CO_2_ | | | | | | | | | | leaves-derived CO_2_ | | | | | | | |
|  | DL1:1 | | DL1:3 | | DL1:5 | | b-DL1:1 | | |  | DL1:1 | | DL1:3 | | DL1:5 | | b-DL1:1 |
| D | 9.517 | | 8.248 | | 12.78 | | 2.894 | | | L | 7.955 | | 6.309 | | 5.652 | | 0.855 |
| DL1:1 |  | | 17.77 | | 22.29 | | 12.41 | | | DL1:1 |  | | 1.646 | | 2.304 | | 8.811 |
| DL1:3 |  | |  | | 4.528 | | 5.354 | | | DL1:3 |  | |  | | 0.657 | | 7.164 |
| DL1:5 |  | |  | |  | | 9.883 | | | DL1:5 |  | |  | |  | | 6.507 |
| Normalised amount of respired CO_2_ | | | | | | | | | | | | | | | | | |
| daphnia-derived CO_2_ | | | | | | | | | | leaves-derived CO_2_ | | | | | | | |
|  | | DL1:1 | DL1:3 | | | DL1:5 | | b-DL1:1 | |  | | DL1:1 | DL1:3 | | DL1:5 | b-DL1:1 | |
| D | | 7.582 | 10.98 | | | 17.7 | | 2.3 | | L | | 6.338 | 1.584 | | 0.5811 | 0.681 | |
| DL1:1 | |  | 3.394 | | | 10.12 | | 9.887 | | DL1:1 | |  | 4.754 | | 5.757 | 7.019 | |
| DL1:3 | |  |  | | | 6.724 | | 13.28 | | DL1:3 | |  |  | | 1.003 | 2.265 | |
| DL1:5 | |  |  | | |  | | 20 | | DL1:5 | |  |  | |  | 1.262 | |
|  | |  | p-value <0.05 | | | | | | |  | |  |  | |  |  | |
|  | |  | p-value >0.05 | | | | | | |  | |  |  | |  |  | |


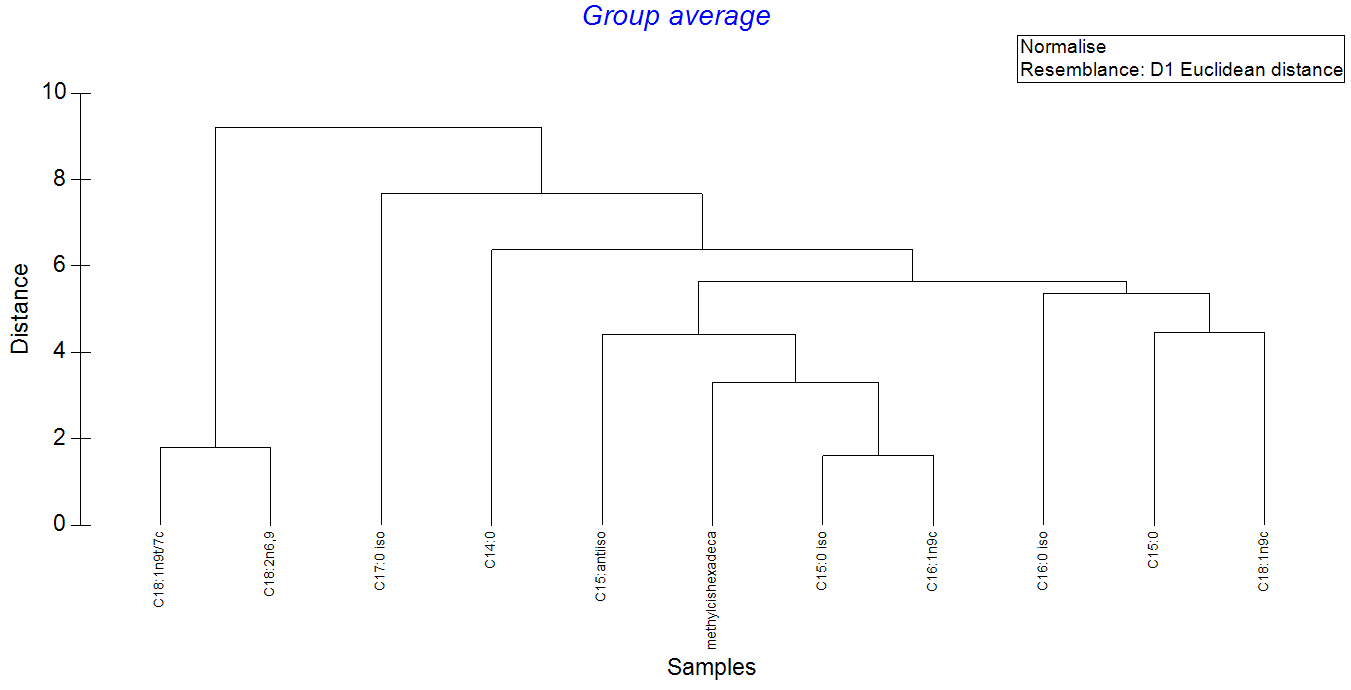


**Supplementary figure 3:** PLFA cluster analysis using euclidean distances of a variables resemblance matrix (PRIMER-e, v.6). The two PLFA of the left cluster (18:1ω9t/7c and 18:2ω6,9) were used as indicators of eukaryotic biomass and the six PLFAs of the right cluster were assigned to bacterial biomass.

**Supplementary table 7:** Summary of two-way ANOVA test (Bonferonni’s multiple comparisons) of eukaryotic and bacterial PLFA concentrations across treatments (**Figure 3**). Numbers correspond to the t‑values and statistical significances are expressed as cell shade (light grey = significant; dark grey = not significant).

| **Eukaryotic PLFA** | | | | | | | | | | | | | | | | | | | | | | | | | | | | | | |
| --- | --- | --- | --- | --- | --- | --- | --- | --- | --- | --- | --- | --- | --- | --- | --- | --- | --- | --- | --- | --- | --- | --- | --- | --- | --- | --- | --- | --- | --- | --- |
| **initials** | | | | | | | | | | | | | | | | **finals** | | | | | | | | | | | | | | |
|  | D | | | DL1:1 | | | DL1:3 | | DL1:5 | | L | | | b-DL1:1 | |  | | | D | | | DL1:1 | | | DL1:3 | | | DL1:5 | L | b-DL1:1 |
| B | 11,53 | | | 9,62 | | | 7,54 | | 4,83 | | 0,26 | | | 13,33 | | B | | | 0,51 | | | 1,35 | | | 1,79 | | | 2,09 | 0,28 | 1,45 |
| D |  | | | 2,59 | | | 4,52 | | 7,04 | | 11,28 | | | 0,86 | | D | | |  | | | 0,83 | | | 1,28 | | | 1,58 | 0,80 | 0,94 |
| DL1:1 |  | | |  | | | 2,08 | | 4,79 | | 9,36 | | | 3,71 | | DL1:1 | | |  | | |  | | | 0,44 | | | 0,75 | 1,63 | 0,11 |
| DL1:3 |  | | |  | | |  | | 2,71 | | 7,28 | | | 5,78 | | DL1:3 | | |  | | |  | | |  | | | 0,30 | 2,07 | 0,34 |
| DL1:5 |  | | |  | | |  | |  | | 4,56 | | | 8,50 | | DL1:5 | | |  | | |  | | |  | | |  | 2,37 | 0,64 |
| L |  | | |  | | |  | |  | |  | | | 13,06 | | L | | |  | | |  | | |  | | |  |  | 1,74 |
| **Bacterial PLFA** | | | | | | | | | | | | | | | | | | | | | | | | | | | | | | |
| **initials** | | | | | | | | | | | | | | | | **finals** | | | | | | | | | | | | | | |
|  | D | | | DL1:1 | | | DL1:3 | | DL1:5 | | L | | | b-DL1:1 | |  | | | D | | | DL1:1 | | | DL1:3 | | | DL1:5 | L | b-DL1:1 |
| B | 7,93 | | | 7,26 | | | 4,73 | | 3,49 | | 0,25 | | | 7,55 | | B | | | 3,72 | | | 10,3 | | | 9,00 | | | 9,05 | 0,73 | 27,8 |
| D |  | | | 1,20 | | | 3,55 | | 4,70 | | 7,70 | | | 0,92 | | D | | |  | | | 6,57 | | | 5,29 | | | 5,33 | 2,99 | 24,1 |
| DL1:1 |  | | |  | | | 2,53 | | 3,77 | | 7,00 | | | 0,29 | | DL1:1 | | |  | | |  | | | 1,28 | | | 1,24 | 9,55 | 17,6 |
| DL1:3 |  | | |  | | |  | | 1,24 | | 4,47 | | | 2,83 | | DL1:3 | | |  | | |  | | |  | | | 0,04 | 8,27 | 18,8 |
| DL1:5 |  | | |  | | |  | |  | | 3,23 | | | 4,07 | | DL1:5 | | |  | | |  | | |  | | |  | 8,32 | 18,8 |
| L |  | | |  | | |  | |  | |  | | | 7,30 | | L | | |  | | |  | | |  | | |  |  | 27,1 |
| **Statistical differences between initial and final PLFA concentrations within same treamtent** | | | | | | | | | | | | | | | | | | | | | | | | | | | | | | |
| **bacterial PLFA** | | | | | | | | | | | | | | | | **eukaryotic PLFA** | | | | | | | | | | | | | | |
| B | D | | DL1:1 | | | DL1:3 | | DL1:5 | | L | | | b-DL1:1 | | | B | | D | | | DL1:1 | | | DL1:3 | | | DL1:5 | | L | b-DL1:1 |
| 0,24 | 5,11 | | 3,10 | | | 4,49 | | 5,91 | | 0,28 | | | 22,23 | | | 0,59 | | 11,87 | | | 8,89 | | | 6,00 | | | 2,54 | | 0,03 | 13,02 |
|  | |  | | | p-value <0.05 | | | | | | |  | | |  | |  | | |  | | |  | | |  | |  |  |  |
|  | |  | | | p-value >0.05 | | | | | | |  | | |  | |  | | |  | | |  | | |  | |  |  |  |

**Supplementary table 8** Fraction of C from single PLFAs derived from either leaves or daphnia material. Calculations are based on a two source mixing model (Equation 1) using PLFA-specific ^13^C isotopic values. Empty cells correspond to samples for which isotopic values could not be measured. **D:L**: daphnia and leaves in a given ratio with a complex microbial community; **b‑DL1:1**: daphnia and leaves (ratio 1:1) with a bacterial community only.

| sample | time | 18:2ω6,9 (eukaryotic) | | 16:1ω9 (bacterial) | | cy-17:0 (bacterial) | |
| --- | --- | --- | --- | --- | --- | --- | --- |
|  |  | leaves-derived | daphnia-derived | leaves-derived | daphnia-derived | leaves-derived | daphnia-derived |
| DL1:1 | initial |  |  |  |  |  |  |
|  | final |  |  | 56 % | 44 % |  |  |
| DL1:3 | initial |  |  |  |  |  |  |
|  | final |  |  | 67 % | 33 % |  |  |
| DL1:5 | initial | 16 % | 84 % | 27 % | 73 % |  |  |
|  | final | 73 % | 27 % | 69 % | 31 % |  |  |
| b-DL1:1 | initial | 3 % | 97 % |  |  |  |  |
|  | final |  |  | 36 % | 64 % | 3 % | 97 % |

**Supplementary table 9:** PERMANOVA analyses of fungal ITS and bacterial 16S data. Pairwise comparisons of treatment effect on final community composition. Statistical significances are expressed as cell shade (light grey = significant; dark grey = not significant).

|  | | ITS | | 16S | |
| --- | --- | --- | --- | --- | --- |
| Pairwise comparison | | t | p-values | t | adj. p-values |
| DL1:1 | DL1:3 | 1,1602 | 0,095 | 1,3807 | 0,034 |
| DL1:1 | DL1:5 | 1,4996 | 0,028 | 1,6429 | 0,032 |
| DL1:1 | D | 1,429 | 0,036 | 1,4127 | 0,03 |
| DL1:1 | L | 1,2744 | 0,037 | 1,8577 | 0,03 |
| DL1:1 | B | 1,6487 | 0,031 | 2,0532 | 0,024 |
| DL1:3 | DL1:5 | 1,0885 | 0,273 | 1,1386 | 0,086 |
| DL1:3 | D | 1,5142 | 0,029 | 1,584 | 0,036 |
| DL1:3 | L | 1,1541 | 0,089 | 1,6684 | 0,03 |
| DL1:3 | B | 1,5996 | 0,021 | 1,9814 | 0,032 |
| DL1:5 | D | 1,8563 | 0,026 | 1,6309 | 0,029 |
| DL1:5 | L | 1,4534 | 0,02 | 1,5701 | 0,023 |
| DL1:5 | B | 1,8463 | 0,026 | 1,8576 | 0,035 |
| D | L | 1,2291 | 0,056 | 1,5277 | 0,036 |
| D | B | 1,2862 | 0,029 | 1,5065 | 0,041 |
| L | B | 1,1248 | 0,148 | 1,1304 | 0,148 |
| b-DL1:1 | DL1:1 |  |  | 3,0344 | 0,026 |
| b-DL1:1 | DL1:3 |  |  | 2,9181 | 0,031 |
| b-DL1:1 | DL1:5 |  |  | 2,9144 | 0,029 |
| b-DL1:1 | D |  |  | 2,1171 | 0,031 |
| b-DL1:1 | L |  |  | 2,6138 | 0,0302 |
| b-DL1:1 | B |  |  | 2,1253 | 0,0282 |


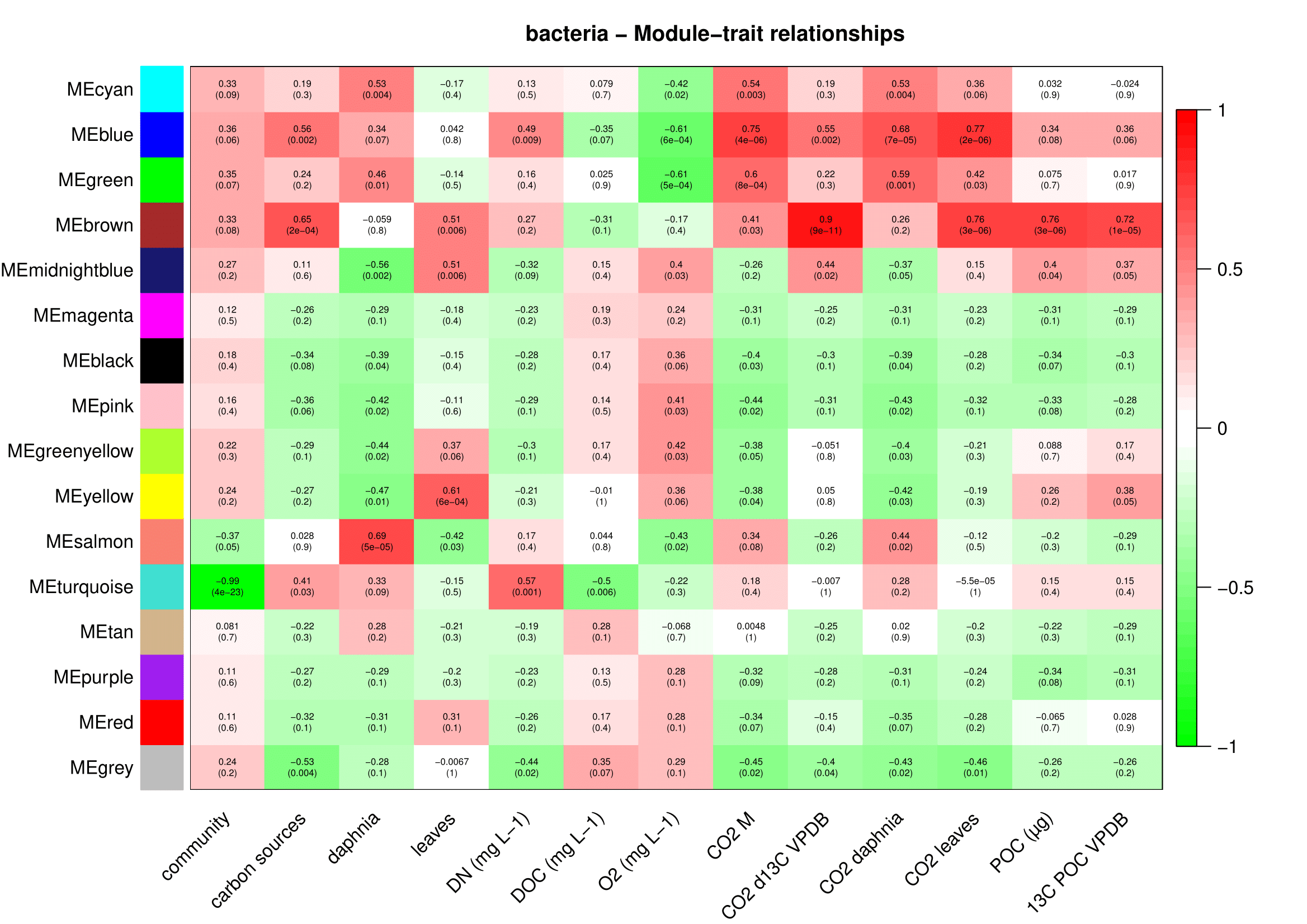
**Supplementary figure 4:** Heatmap showing WGCNA modules and their correlations to external traits. Upper numbers of each cell correspond to correlation coefficients and lower numbers in brackets to the statistical significance. We extracted OTUs of the blue, green and brown module, as these were correlated to either “daphnia-derived CO_2_” (green), “leaves-derived CO_2_” (brown) or both (blue). The variables used were: **community** (complex vs. bacterial), **carbon sources** (single (D and L controls) vs. mixed), **daphnia** (amount of daphnia (mg) added), **leaves** (amount of leaves (mg) added), **DN**, **DOC**, **O_2_**, **CO_2_** (total CO_2_ concentrations), **CO_2_ d^13^C** (total ^13^CO_2_ fraction), **CO_2_ daphnia/leaves** (calculated daphnia/leaves-derived CO_2_), **POC** and total **^13^C** fraction of POC.
